# Constructing the spatiotemporal atlas of single-cell lineage trajectories in stereotypic biological structures

**DOI:** 10.1016/j.isci.2025.114307

**Published:** 2025-12-01

**Authors:** Ran Wang, Xianfa Yang, Chengfei Lian, Jianjie Wang, Jiehui Chen, Yun Qian, Yaochen Xu, Liantang Wang, John C. Marioni, Patrick P.L. Tam, Naihe Jing

**Affiliations:** 1 Key Laboratory of Biomedical Information Engineering of Ministry of Education, Biomedical Informatics & Genomics Center, School of Life Science and Technology, Xi’an Jiaotong University, Xi’an, Shaanxi 710049, China; 2 State Key Laboratory of Cell Biology, CAS Center for Excellence in Molecular Cell Science, Shanghai Institute of Biochemistry and Cell Biology, Chinese Academy of Sciences, University of Chinese Academy of Sciences, 320 Yue Yang Road, Shanghai 200031, China; 3 Guangzhou National Laboratory, Guangzhou 510005, China; 4 School of Mathematics, Northwest University, Xi’an 710127, China; 5 Cancer Research UK Cambridge Institute, University of Cambridge, Cambridge CB2 0RE, UK; 6 European Molecular Biology Laboratory, European Bioinformatics Institute (EMBL-EBI), Cambridge CB10 1SD, UK; 7 Embryology Research Unit, Children’s Medical Research Institute, University of Sydney, Camperdown, NSW, Australia; 8 School of Medical Sciences, Faculty of Medicine and Health, University of Sydney, Camperdown, NSW, Australia

**Keywords:** Developmental biology, Computational bioinformatics, Transcriptomics

## Abstract

Spatial transcriptomics technologies have emerged as instrumental tools for elucidating cellular heterogeneity and molecular regulation within the complex tissue microenvironment, but are constrained by insufficient gene recovery or an inability to achieve intact single-cell resolution. By integrating spatial transcriptomics and single-cell RNA sequencing technologies, we develop a mathematical method of single-cell resolved spatiotemporal (SCST) mapping that comprises tiered algorithms for constructing the spatial molecular atlas of the biospecimen at single-cell resolution across a timeline of development. The embedded spatial-smoothing algorithm in SCST significantly enhances the spatial mapping accuracy of single cells, thereby improving the fidelity of the annotation of cell identity to the equivalent *in vivo* cell type. Through 3D mathematical modeling, SCST facilitates the spatial reconstruction of a single-cell molecular atlas and the delineation of cellular heterogeneity. When integrated with temporal data, SCST can also delineate the spatiotemporal lineage trajectory at single-cell resolution in a developing biological entity.

## Highlights

• A single-cell resolved spatiotemporal (SCST) mapping methodology was developed
• SCST significantly enhances the spatial mapping accuracy of single cells
• SCST reconstructs the 3D single-cell atlas and delineates the spatial heterogeneity
• SCST delineates the spatiotemporal lineage trajectories at single-cell resolution

## Graphical abstract

**Figure:**
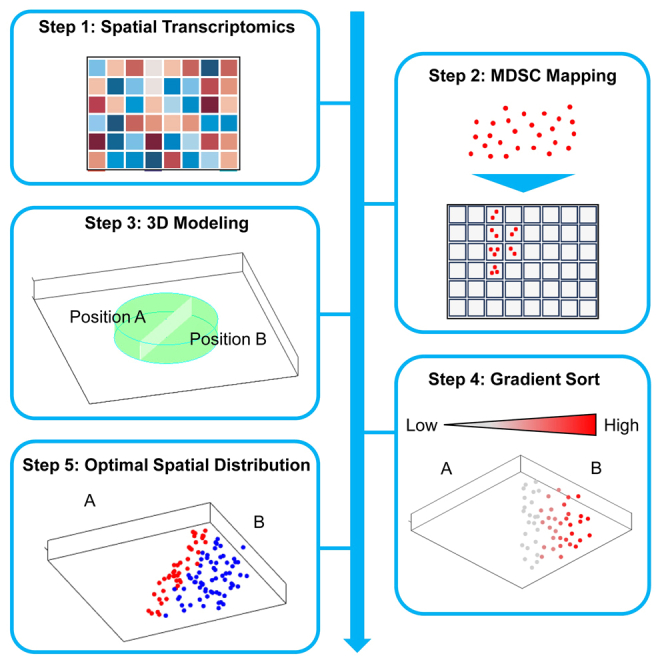

## Introduction

Emerging spatially resolved transcriptome technology has provided useful insights into the complexity of cell types in embryonic tissues and the transcriptional activity that guides lineage differentiation and tissue patterning during embryonic development.^1^^,^^2^^,^^3^ For example, seqFISH characterized the spatial patterning of cells within the gut tube at the E8.75 mouse embryo in a two-dimensional (2D) section.^4^ DBiT-seq was applied to tissue sections of an E10 mouse embryo to anatomically define the major tissue regions.^5^ However, current spatial transcriptomic methodologies remain constrained by two fundamental limitations: insufficient sequencing depth for comprehensively profiling the transcriptomes of precisely localized single cells, and an inability to achieve whole-tissue coverage, thereby failing to reconstruct tissue architecture in its entirety across three dimensions. These technical shortcomings critically impede our ability to elucidate the molecular mechanisms underlying cell fate determination, lineage specification, and spatial patterning during tissue morphogenesis. Recent application of the sampling of low cell-number or single cell populations coupled with high-throughput sequencing techniques for transcriptome and further at multi-omics layers offer the opportunity to gain information of the molecular attributes of cell fate choice and cell identity at single-cell resolution.^6^^,^^7^^,^^8^ Beyond the cell autonomous molecular activity, cell fate choices and diversification of cell types are also influenced by the interactive signals perceived by the cells and other niche factors at a specific spatial location.^9^ Single-cell RNA-sequencing (scRNA-seq) approaches have been used to profile the molecular features of individual cells, but most scRNA-seq technologies require tissue dissociation, resulting inevitably in the loss of spatial information related to tissue architecture and organization.^3^ With the increasing sampling of cells in numbers and cell types from a comprehensive coverage of tissue domains of the biospecimen, a fully spatial transcriptome could be constructed. However, often this would necessitate the re-sampling of the biospecimens across space to build such a molecular cell atlas.

To harness the valuable resource of pre-existing scRNA-seq datasets, we developed a mathematical method of **s**ingle-**c**ell resolved **s**patio**t**emporal (SCST) mapping that comprises tiered algorithms for constructing the single-cell resolved spatial molecular atlas of the biospecimen across a timeline of development. Embedded in SCST, the multi-dimensional single cell mapping algorithm (MDSC Mapping V2), which incorporates the digitalized spatial reference transcriptome data as a positioning system with 3D coordinates and an allied spatial-smoothing process, has enhanced the accuracy of spatial mapping of single cells, and improved the fidelity of the annotation of the cell identity of the mapped cells to the equivalent *in vivo* cell type. Together, mathematical modeling and the Optimal Spatial Distribution algorithm facilitate the spatial reconstruction of a single-cell molecular atlas and the delineation of cellular heterogeneity at defined spatial domains.

Applying SCST to a single-cell dataset of gastrula-stage mouse embryos, “Gastrulation Atlas,”^10^ we demonstrated the utility of the mapping method in generating a high-dimensional atlas of space- and stage/time-resolved gene expression profiles of single cells in the germ layers of the gastrulating mouse embryos, which display stereotypic morphology at each developmental stage. By implementing a Digital Lineage Tracing algorithm for integrative analysis of single-cell gene expression profiles with spatial registry and time stamps, the transition of cell states in the developmental trajectory can be reconstructed by computing transcriptomic similarities between single cells, thereby enabling the modeling of the spatiotemporal developmental trajectory at the single-cell resolution of the successively diversifying cell types in the germ layers during mouse gastrulation.

## Results

### Overview of the single-cell resolved spatiotemporal mapping methodology

**Figure 1:**
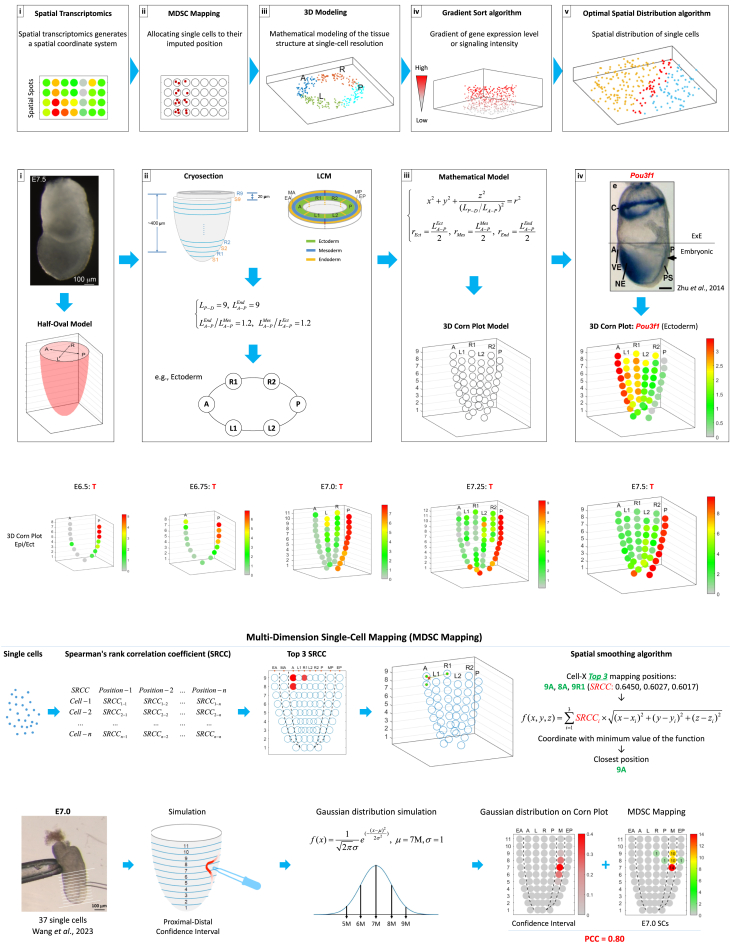
Single-cell resolved spatiotemporal (SCST) mapping protocol and multi-dimension single-cell (MDSC) mapping (A) A flow diagram of the 5-step SCST protocol. (i) Spatial transcriptomics generates a spatial coordinate system of the target tissue. (ii) MDSC mapping allocates single cells to their imputed position. (iii) 3D Modeling simulates the spatial positions. Single cells that mapped to a specific spatial position were distributed uniformly across the interior space of the position in each 3D model. (iv) Gradient Sort algorithm displays the cells in relation to the gradient of gene expression level or signaling intensity. (v) Optimal spatial distribution algorithm refines the spatial distribution pattern of cell types by optimal coordinates at each spatial position. (B) Modeling of the embryo and visualization of Geo-seq data. (i) A half-oval model mirrors the architecture of the cup-shaped gastrula-stage mouse embryo. (ii) The Geo-seq cell samples are distributed uniformly on the cross-section of the half-oval model. The formulae summarize the parameter settings of the E7.5 embryo model. *L*_*P*-*D*_ represents the length of the proximal-distal axis of the half-oval model of the embryo, ${\;L}_{A-P}^{Ect}$, $L_{A-P}^{Mes}$ and $L_{A-P}^{End}$ represent the diameters of the half-oval model of the ectoderm, mesoderm and endoderm layer (See the detail parameter settings in Figure S1B). (iii) Integration of all sections to generate the 3D corn plot model. The formulae recapitulate the half-oval model. (iv) The exemplar 3D corn plot shows the spatial pattern of expression of *Pou3f1* in the ectoderm layer of the E7.5 embryo. WISH image was sourced from a previous study by Zhu et al.^11^ (C) 3D corn plots show the spatiotemporal pattern of expression of *T* in the epiblast/ectoderm layer of E6.5-E7.5 embryos. The proximal-distal span of the *T*+ domain in the posterior epiblast defines the developmental stage of the gastrulating embryo. The color legend indicates the level of expression determined by transcript counts. (D) The pipeline of multi-dimension single-cell (MDSC) mapping. The SRCCs of the expression values of the zipcodes of every single cell against all reference samples of the reference embryo were calculated, followed by the application of a spatial smoothing algorithm to impute the high-confidence (closest) location (STAR Methods). The mapping of cells to position 9A of the E7.5 embryo was shown as an example. (E) Verification of the results of MDSC mapping of single cells isolated from a known position in the E7.0 embryo. Image processing and Gaussian distribution were applied to mathematically simulate the confidence intervals. Pearson correlation coefficient (PCC) between the confidence intervals and MDSC Mapping results was calculated to evaluate the accuracy of the mapping methodology. The number on corns represents the number of cells mapped to the designated Geo-seq position in the germ layers. PCC values and confidence intervals are shown for the simulation. The image of isolating single cells was from a previous study by Wang et al.^12^ Abbreviations: Epiblast/Ectoderm - A, anterior; P, posterior, R, right; R1, right anterior, R2, right posterior; L, left; L1, left anterior; L2, left posterior; Mesoderm - M, mesoderm; MA, anterior mesoderm; MP, posterior mesoderm; Endoderm - EA, anterior endoderm; EP, posterior endoderm. See also Figures S1–S3 and Table S1.

The SCST workflow is composed of five steps: (1) We first use spatial transcriptomics technology to establish a spatial coordinate system of the target tissue. In this study, we employed the geographical position sequencing (Geo-seq) technology (Figure 1Ai).^12^^,^^13^ (2) In combination with the coordinate system and scRNA-seq datasets, MDSC Mapping accurately infers the spatial position of the cells (Figure 1Aii). (3) Then we apply 3D modeling to simulate the specific spatial positions. At this step, single cells that mapped to a specific spatial position were distributed uniformly across the interior space of the position in each 3D model (Figure 1Aiii). (4) Apply the Gradient Sort algorithm, which is based on the gradient of vectorially graded molecular activity (gene expression level and intensity of signaling response), to refine the spatial distribution of the single cells in the 3D geometric model (Figure 1Aiv). (5) Finally, apply the Optimal Spatial Distribution algorithm to assign the optimal coordinates for the single cells to delineate the heterogeneity of cell population at each spatial position and to construct the spatiotemporal molecular trajectory of single-cell populations (Figure 1Av).

Each step of the tiered algorithms of SCST can be applied independently in a spatial omics study for different research objectives, including but not limited to mapping single cells spatially to a biological structure, revealing the high-confidence spatial distribution pattern of single cells, annotating the equivalent *in vivo* cell identity, and charting the spatiotemporal developmental trajectory of cell lineages.

### Generating a reference set of positional addresses in a stereotypical biological structure

A key prerequisite for the construction of the molecular cell atlas of spatiotemporal trajectories in the biospecimen is the collation of a stage- and spatially registered population-based transcriptome dataset. In previous studies, we applied Geo-seq to collate the spatially resolved transcriptome of cell populations at defined positions in the germ layers of gastrulation stage embryos (Figure S1A).^12^^,^^13^ The first step of SCST is to replicate the spatial architecture of the embryo and visualize the spatial pattern of gene expression, we devised a geometric model (the “half-oval” model) that recapitulates the morphology of the germ layers in the gastrula-stage mouse embryo (Figure 1Bi; STAR Methods), with each cell population assigned a spatial coordinate as the positional address (Figures 1Bii, 1Biii, and S1B). For data visualization, the spatiotemporal transcriptome data were rendered digitally for depiction in the “3D corn plot” model. For example, the *Pou3f1-*expressing cell population is mapped to the anterior ectoderm of the E7.5 embryo (Figure 1Biv), and the proximal-distal span of the *T-*expressing cell population is mapped to the posterior epiblast that contains the primitive streak during the course of gastrulation (Figures 1C and S2A).

### Multi-dimension single-cell mapping

While an extensive compendium of single-cell transcriptome datasets has been collated, the utility of these datasets for constructing the spatiotemporal transcriptome of a biological entity has been constrained by the lack of positional register of cells, which is lost by sampling cells after tissue dissociation, and the gaps in the coverage across developmental timepoints. A high-value attribute of the 3D corn plot model is establishing a reference spatial positioning system. From the spatial transcriptome of cell populations, sets of position-specific transcripts (designated as the zipcode genes, zipcodes for short), are captured for embryos at five timepoints of gastrulation (Figure S2B; Table S1; STAR Methods). The information of positional address of the 3D corn plot model in combination with zipcodes is utilized as a basis for the spatial mapping of single cells. Utilizing SCST, the embedded multi-dimension single-cell mapping algorithm (MDSC mapping, version 2, see STAR Methods for the operation procedure and updates) (Figure 1D) was applied to map the cells to their best inferred position in the embryo. To evaluate the precision of positional mapping, we mapped single cells sampled from known positions in embryos at five developmental timepoints of gastrulation. The results showed that the single cells could be mapped at high confidence to their site of origin (Figures 1E, S3A, and S3B; STAR Methods).

Previous works of mapping the location of cells in biological structures on the basis of the concordance of the gene expression profile have been confounded by mathematical uncertainties and false-positives,^2^^,^^6^^,^^14^^,^^15^ especially spatial “landmark” genes are barely expressed at early developmental stages.^3^^,^^16^ By incorporating a spatial-smoothing algorithm into the spatial positioning system, the mapping efficiency was significantly enhanced (Figures 1D and 1E; STAR Methods). For comparison, we applied other single-cell mapping methodologies for the test-samples of single cells, including CytoSPACE mapping,^17^ Seurat mapping,^18^ and mutual nearest neighbors (MNN) mapping^19^ (Figures S3A and S3B). The benchmarking results indicated that MDSC mapping has achieved a higher level of accuracy than other methods. MDSC mapping shows technical superiority when the sample size is small, and there is an absence of spatial “landmark,” especially at the early development stages. The embedded spatial-smoothing algorithm significantly improves the mapping fidelity.

**Figure 2:**
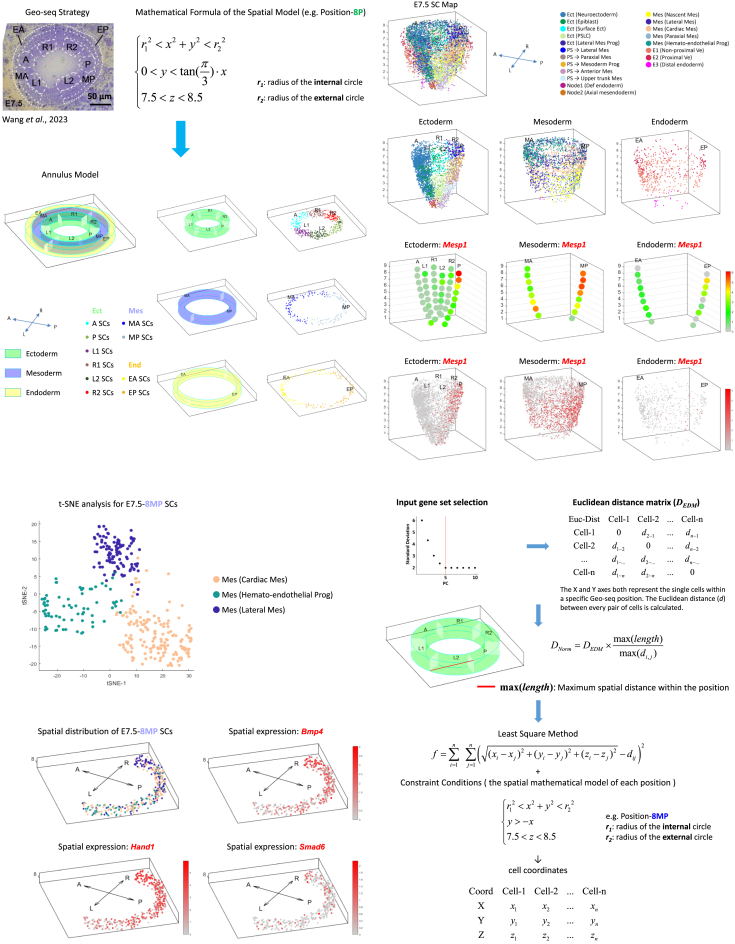
3D modeling for the single-cell resolution embryo map (A) Annulus Model replicates the spatial organization of the germ layers for the display of the distribution of single cells. Single cells that mapped to a Geo-seq position were distributed uniformly across the corresponding interior space of each domain in the annular section. The image of Geo-seq of cell samples was from a previous study by Wang et al.^12^ Scale bars, 50 μm. (B) Single-cell (SC) maps showing the spatial distribution of single cells identified in the “scGastrulation” of E7.5 mouse embryo (top panel). Cells are colored by their cell-type annotation (see STAR Methods for nomenclature). The SC maps in the three germ layers (ectoderm, mesoderm and endoderm) are shown separately (bottom panels). Abbreviations: Ect – ectoderm; PS – primitive streak; PSLC – primitive streak like cells; Mes – mesoderm; E − endoderm; Ve – visceral endoderm; Def – definitive; Prog – progenitor. (C) 3D corn plots (top) and SC maps (bottom) showing the spatial distribution of *Mesp1*-expressing cells in the three germ layers of the E7.5 embryo. The color legend indicates the level of expression determined by the transcript counts. (D) *t*-distributed stochastic neighbor embedding (*t*-SNE) plot showing the single cells in position-8MP of E7.5 embryo. (E) The optimal spatial distribution algorithm for re-ordering the spatial distribution of single cells within a Geo-seq position by Euclidean-distance derived optimal coordinates (STAR Methods). (F) The imputed spatial distribution of single cell types within position-8MP of the E7.5 embryo (top-left panel, cell types are annotated as in Figure 2D) and the spatial distribution of *Bmp4-, Hand1-, and Smad6*-expressing cells. The color legend indicates the level of expression determined by the transcript counts. See also Figures S4–S6.

Applying the MDSC mapping algorithm, we mapped the single cells of the “Gastrulation Atlas” onto the germ layers of the E6.5-E7.5 embryos.^10^^,^^12^ To visualize the spatial distribution of the cells in the germ layers, we displayed the cells on geometric models (Figures 2A and S4A–S4C; STAR Methods) in a series of 3D-rendered spatiotemporal maps. After allocating single cells to the geometric model, particularly the annulus model, we applied mathematical modeling and developed a Gradient Sort algorithm to rearrange single cells within each Geo-seq position by incorporating the information of vectorially graded molecular activity (e.g., *Bmp4* expression) (Figure S4D; STAR Methods) that may reflect the regionalization of cell types in a specific tissue domain. This mathematical model enables an effective imputation of the coordinates of single cells at the Geo-seq position (Figure S4E). By the integration of all the annular domains, the single-cell resolution molecular cell atlas was constructed. For example, at E7.5, the molecular map displays the distribution of the full range of cell types in the three germ layers (Figures 2B and S5A; see STAR Methods for nomenclature), such as the distribution of “Node1 (Def endoderm)” cells in the distal ectoderm and “Mes (Cardiac Mesoderm)” cells in the proximal mesoderm (Figure S5A). This single-cell 3D rendition also enables the visualization of the whereabout of cells displaying specific gene expression pattern (Figures 2C and S5B), for examples, *Mesp1*-and *Mixl1*-expressing cells are mapped primarily to the posterior epiblast/primitive streak and the mesoderm (Figures 2C and S5B), *Otx2*-expressing cells and *Sox17*-expressing cells mostly in the anterior ectoderm and the endoderm respectively (Figure S5B).

### Optimal spatial distribution algorithm reveals cell type heterogeneity in Geo-seq defined population

To simulate the spatial distribution of cell types within each Geo-seq position (or other biological structures), an Optimal Spatial Distribution algorithm based on Euclidean distance was developed (Figures 2D–2F; STAR Methods). We made the assumption that cells that are physically close tend to share similar transcription profiles, and vice versa. Using the gene-expression matrix of single cells mapped to a specific position as an input, computing a normalized Euclidean distance matrix, followed by applying the least square method under specific constraint conditions (the spatial mathematical model of the corresponding position) enabled the assignment of an optimized coordinate to every single cell (Figure 2E). For example, we applied this algorithm to simulate the spatial distribution pattern of single cells at position-8MP of the E7.5 embryo (Figures 2D and 2F). In accordance with the previous report,^12^ the left-right (L-R) body asymmetry of the embryo in the proximal mesoderm was clearly depicted by the asymmetric pattern of right-side enhanced expression of *Bmp4*, *Hand1,* and *Smad6* (Figures 2F and S6A–S6C).

To benchmark the comparative performance of Optimal Spatial Distribution algorithm, we also used CSOmap^20^ and novoSpaRc^21^ to simulate the spatial distribution pattern of single cells of specific domains. CSOmap employs a ligand-receptor interaction-based embedding to infer the spatial positions of cells within a 3D environment. novoSpaRc is an optimal-transport based method that *de novo* spatially reconstructs the single-cell gene expression in a 2D space. The results showed that at position-8MP of the E7.5 embryo, neither CSOmap nor novoSpaRc could detect the spatial heterogeneity among different cell types (Figures S6D and S6E). Thus, the Optimal Spatial Distribution algorithm can be exploited to identify the organizational principle for the spatial expression of genes in embryonic tissues and to infer meaningful probabilities of spatial position for individual cells. This algorithm is an independent functional module of SCST mapping, it is compatible with any single-cell technology and can be applied in any tissue with or without a stereotypic biological structure (through adjusting the constraint condition of the Least Square Method).

### A single-cell resolution spatiotemporal molecular atlas

**Figure 3:**
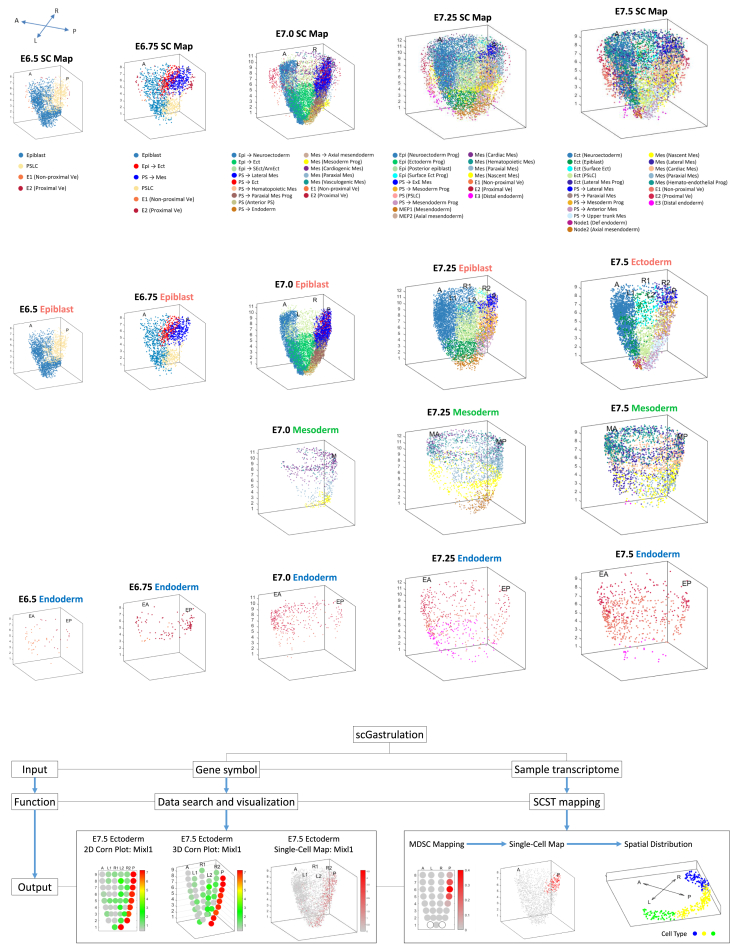
The spatiotemporal distribution of single cells in E6.5-E7.5 mouse embryos (A) The spatiotemporal distribution of the full range of cell types identified in the E6.5-E7.5 mouse embryos (data available in “scGastrulation,” http://scgastrulation.sibcb.ac.cn). Color codes represent cell-type annotation (see STAR Methods for nomenclature). Abbreviations: Epi – epiblast; Ect – ectoderm; PS – primitive streak; PSLC – primitive streak like cells; Mes – mesoderm; E − endoderm; MEP – putative mesendoderm progenitors; Ve – visceral endoderm; Def – definitive; Prog – progenitor. (B) The spatiotemporal distribution of single cells in the epiblast/ectoderm, mesoderm, and endoderm of E6.5-E7.5 mouse embryos. (C) The functionalities of the “scGastrulation” web portal: Data search and visualization in a 2D corn plot, a 3D corn plot, or a single-cell molecular map format, and the workflow of the SCST mapping.

Taking into consideration the pattern of cell distribution across the developmental stages, the data could be rendered digitally into the spatiotemporal (4D) Atlas at single-cell resolution (Figures 3A and 3B). This 4D Atlas provides unique insights into the spatial distribution of single cells of specific lineages in the germ layers of E6.5-E7.5 embryos, thus reflecting the progressive diversification of cell types in the germ layers during gastrulation.

To avail the space-, time- and single-cell resolved transcriptome data as a resource to the scientific community, a web portal, “scGastrulation” at http://scgastrulation.sibcb.ac.cn has been established to provide open access of the spatiotemporal transcriptome data. The “scGastrulation” offers (1) data search functionality: querying and displaying the spatiotemporal expression pattern of genes of interest in a 2D corn plot, a 3D corn plot or a single-cell molecular map format; (2) data processing functionality: a step-by-step workflow of the SCST mapping method (Figure 3C).

### Single-cell resolved spatiotemporal mapping reveals the spatiotemporal trajectory of mesodermal lineages during gastrulation

**Figure 4:**
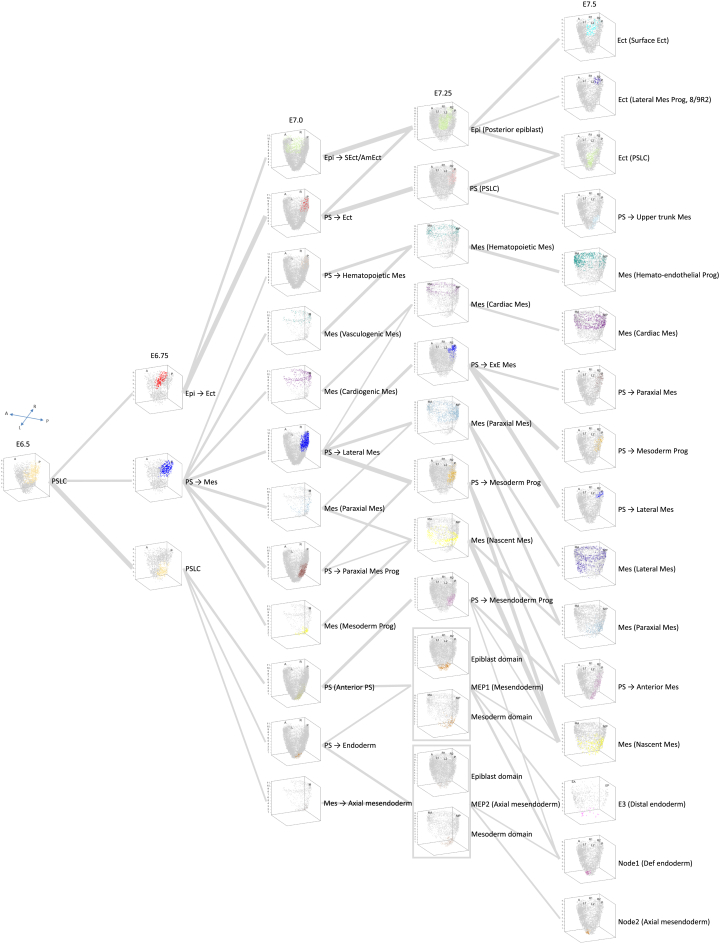
The spatiotemporal developmental trajectory of primitive-streak like cells The spatiotemporal molecular trajectory of the descendants of primitive-streak like cells (PSLCs) from the E6.5 posterior epiblast in the germ layers of E6.75, E7.0, E7.25, and E7.5 embryo. The width of the connector indicates the strength of correlation between the connected cell types. See also Figures S7 and S8.

To reveal the developmental trajectory and the lineage diversification of the cell population, we formulated a shortest-distance hypothesis that cells along a developmental lineage have transcriptional profiles that are often more similar than cells that have distant or no developmental relationship. Based on this premise, we devised a Digital Lineage Tracing algorithm (Figure S7A; STAR Methods) to infer the developmental trajectory of the single cells in the posterior epiblast of E6.5 embryo to cells in the germ layers of embryos at advancing stages of gastrulation (Figure 4). For example, the collation of all the lineage trajectories of E6.5 primitive-streak like cells (PSLCs) showed that from the three germ layer progenitors established by E6.75 (“Epi→Ect,” “PS→Mes,” “PSLC” cells), with a subset of cells in the proximal-posterior epiblast contributes to epiblast/ectoderm lineage, while the bulk of PSLCs is partitioned into proximal and distal cell groups that are allocated to mesoderm and (putative) mesendoderm progenitors respectively (Figure 4). We selected the lineage-specific markers *Pou3f1*, *T,* and *Foxa2* for the three cell types in the posterior epiblast of the E6.75 mouse embryo and performed RNAscope analyses. The results showed the presence of *Pou3f1*-expressing cells (“Epi→Ect” lineage) and *T*-expressing cells (“PS→Mes” lineage) in the proximal posterior epiblast (Figure S7B). The “PSLC” cells destined for the mesendoderm lineage were validated by *Foxa2* expression in the distal posterior epiblast (Figure S7C). Collectively, these results demonstrate that the SCST method enables accurate detection of the cell fate decision process and a more precise definition of cell types.

It is worth noting that the mesoderm progenitors among the PSLCs at E6.5 give rise to multiple types of mesoderm cells during gastrulation, with the early allocation of the blood lineage and *Tal1*-expressing cells from the proximal primitive streak/mesoderm population at E7.0 (Figures 4 and S8A–S8C). Along the molecular trajectory, the spatiotemporal development of mesodermal lineages during gastrulation is clearly depicted. These findings support that the determination and regionalization of cell fates in the germ layers are evident at the molecular level as early as the mid-gastrula stage. These single-cell resolved lineage trajectories so constructed further highlighted the intricate and dynamic molecular control of the specification of cell fates during germ layer development.

### Annotation of cell identity

**Figure 5:**
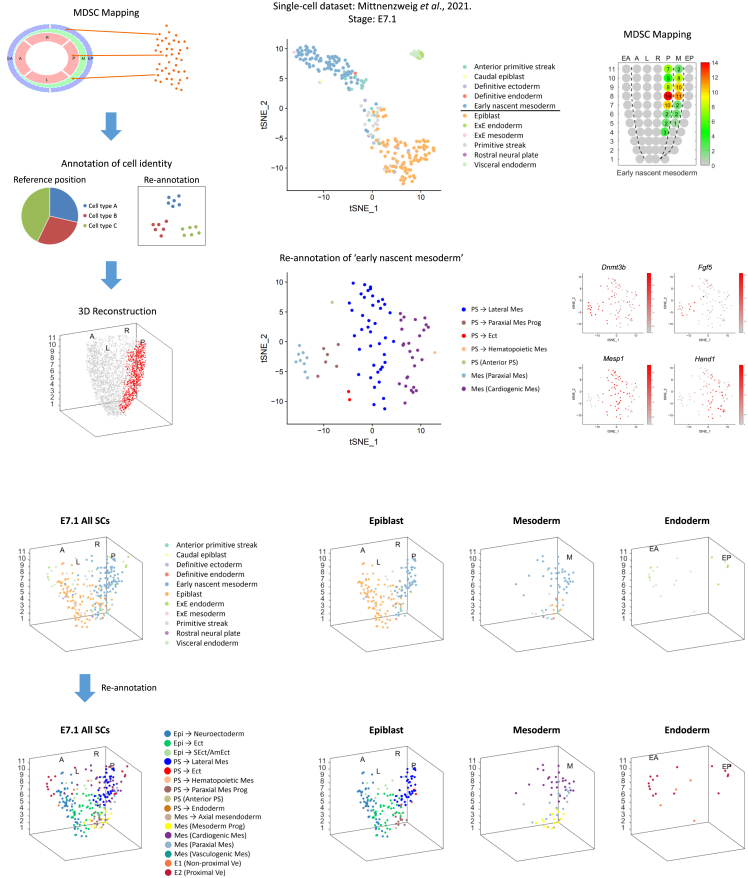
Annotation of cell identity (A) Schematics of the annotation of cell identity of the single cells mapped to specific positions in the germ layers. (B) *t*-SNE plot shows the data structure of a published single-cell dataset (E7.1 mouse embryo, Mittnenzweig et al.^22^). Cell types are annotated (see color legend). (C) Results of MDSC mapping of “early nascent mesoderm” of E7.1 mouse embryo. (D) *t*-SNE plot shows the results of re-annotation of “early nascent mesoderm” cells. Color legend showing the re-annotated cell types. (E) *t*-SNE plots show the expression of representative marker genes of the re-annotated cell types. Mes (Paraxial Mes): *Dnmt3b*, PS→Paraxial Mes Prog: *Fgf5*, PS→Lateral Mes: *Mesp1*, Mes (Cardiogenic Mes): *Hand1*. (F) Modeling the spatial distribution of single cells in the germ layers of the E7.1 mouse embryo. Upper panels: the distribution pattern of single cells with the original cell identity; lower panels: the distribution pattern of single cells with the re-annotated cell identity. The single-cell spatial maps in the three germ layers are also shown separately. See also Figures S9–S12.

An additional attribute of the SCST method is the annotation of the identity of the mapped cells to the equivalent *in vivo* cell type and the delineation of the heterogeneity of cell types in the population at each Geo-seq position (Figure 5A). To illustrate the amenability of annotation of cell types, the set of single cells that were mapped to the inferred positions in the validation experiments (Figure S3A) was subjected to clustering analysis to determine if the assigned cell identity matches that of cells in the *in vivo* domain. The pilot results showed that, without any prior knowledge, the identity of the mapped cells can be annotated with reference to the molecular signature of the cell types in the best-fit domain (Figures S9A–S9D; STAR Methods). For example, the single cells isolated from the posterior mesoderm of E7.0 embryo were principally annotated as Mes (Cardiogenic Mes) and Mes (Paraxial Mes) (Figures S9A–S9C).

To further demonstrate the utility of the mapping method for annotating cell identity and constructing a spatial molecular atlas, we mapped a set of single cells sampled from individual mouse embryos at three different timepoints (E6.5, E7.1, and E7.5) of gastrulation, with the scRNA-seq data pre-processed by metacell analysis.^22^ MDSC mapping was performed with the spatial references of cell populations of the E6.5, E7.0 and E7.5 embryos in our dataset (Figures 5B, 5C, and S10A–S10F), followed by the re-annotation of cell identity based on the single-cell spatial atlas of the gastrulating mouse embryo we established (Figures 3A, 5D, 5E, S12A, and S12B). For example, the cells that are designated as “early nascent mesoderm” were mapped to both the posterior epiblast and the mesoderm of the E7.0 embryo (Figures 5B and 5C), whereas clustering analysis re-annotated these cells as distinct cell types of the primitive streak and mesoderm derivatives (Figures 5D and 5E). To validate the annotation result, we performed RNAscope analysis to determine the spatial distribution of *Mesp1*, a nascent mesoderm marker.^23^ The result showed that *Mesp1* is expressed in the posterior epiblast cells in E7.0 embryo (Figures S11A and S11B). These cells that were previously annotated as “early nascent mesoderm” might represent progenitor cells at a transition state from epiblast to mesoderm. Based on the spatial heterogeneity, the SCST method accurately annotated the cell identity.

Based on the re-annotated cell identities, we reconstructed the series of single-cell resolved spatial molecular maps (Figures 5F, S12A, and S12B), which depict the spatial distribution pattern of single cells of a range of lineages in the germ layers. The resultant spatiotemporal atlas illustrates the differentiation of the ensemble of embryonic cells across three timepoints of gastrulation, and lends support to the inference that gastrulation is dominated by progenitor states that continuously multi-furcate into separate lineages.^22^

### Application of single-cell resolved spatiotemporal mapping in another biological system

**Figure 6:**
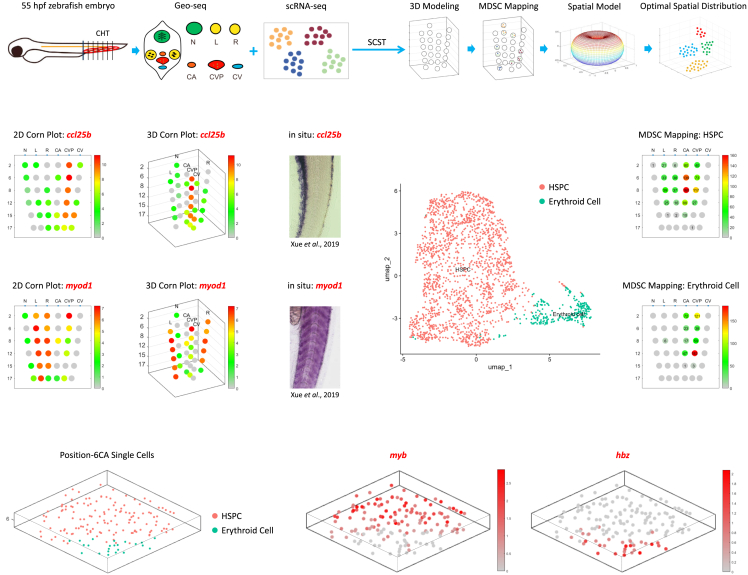
Application of SCST (A) Application of the SCST method in another biological system. SCST reconstructed the spatial architecture of the caudal hematopoietic tissue (CHT) of a 55-hpf zebrafish embryo at single-cell resolution. The six Geo-seq regions include neuro (N), left muscle (L), right muscle (R), caudal artery (CA), caudal vein (CV), and caudal vein plexus (CVP). (B) 2D corn plot model for the Geo-seq data, the spatial gene expression of *ccl25b* (top panel) and *myod1* (bottom panel) was shown as an example. (C) 3D corn plot model for the Geo-seq data, the spatial gene expression of *ccl25b* (top panel) and *myod1* (bottom panel) was shown as an example. (D) Whole mount *in situ* hybridization of *ccl25b* (top panel) and *myod1* (bottom panel). Images were obtained from *Xue* et al.^24^ (E) UMAP plot shows the hematopoietic stem and progenitor cells (HSPCs) and erythroid cells in the zebrafish CHT. (F) MDSC mapping results of HSPCs and erythroid cells. The number in each corn indicates the number of cells mapped to the specific Geo-seq position. (G) The imputed spatial distribution of HSPCs and erythroid cells within position-6CA (left panel) and the spatial gene expression pattern of exemplar marker genes of HSPCs and erythroid cells (*myb* and *hbz*, right panel). See also Figure S13.

The SCST method can also be applied in other systems. Using Geo-seq and scRNA-seq, Xue et al. explored the hematopoietic stem and progenitor cell (HSPC) expansion in the caudal hematopoietic tissue (CHT) of the zebrafish embryo (Figures 6A and S13).^24^ Applying SCST method, we constructed a 3D model for the CHT and simulated the spatial distribution pattern of single cells at specific positions (Figure 6A).

Based on the Geo-seq strategy for the CHT (Figure S13), we first constructed the 2D/3D Corn Plot Models that recapitulate the spatial structure of the CHT (Figures 6B–6D). scRNA-seq identified HSPCs and erythroid cells (Figure 6E). To uncover the spatial localization of HSPCs, MDSC Mapping was applied; the HSPCs were mainly mapped to the caudal artery (CA) and caudal vein plexus (CVP) regions, the erythroid cells showed a similar spatial distribution pattern and were more enriched in the CVP region (Figure 6F). Then we used the Optimal Spatial Distribution algorithm to simulate the spatial distribution of HSPCs and erythroid cells at position-6CA; the two cell types were clearly separated (Figure 6G). Overall, the SCST method shows great generalizability to other species and would be an enabling tool for research in single-cell spatial transcriptomics and further at the multi-omics level.

## Discussion

Single-cell transcriptomics has empowered the identification of cell identity and the tracking of transitions of cell states, but the conventional approach is unable to capture the spatial context of the single cells to glean information on the patterning of cell populations in the embryo during development.^2^^,^^3^^,^^25^ Here, we present SCST, an integrated mathematical model that comprises tiered algorithms to construct the 3D tissue molecular architecture at single-cell resolution, and apply it to generate a single-cell resolved spatiotemporal molecular atlas of mouse gastrulation. The SCST method that leverages the attributes of the existing single-cell datasets and spatial transcriptomics enables the elucidation of the spatiotemporal regionalization of the single-cell population and the heterogeneity of cell types within a specific spatial domain. Each step of SCST can be applied independently in a spatial omics study for different research objectives. The MDSC Mapping attains a higher level of accuracy compared with other single cell spatial mapping methodologies. With digitalized spatial coordinates embedded in the spatial transcriptome data, the allied spatial-smoothing algorithm has significantly improved the mapping fidelity. To simulate the spatial distribution pattern of single cells within a tissue domain, we applied the Optimal Spatial Distribution algorithm to assign an optimized spatial coordinate for every single cell. This mathematical simulation further revealed the spatial heterogeneity of tissue organization at single-cell resolution. In combination with the Digital Lineage Tracing algorithm, it enables the reconstruction of the spatiotemporal (4D) lineage trajectory, based on time-stamped spatial transcriptome data. The resultant molecular cell atlas offers an avenue to glean insights into the molecular attributes and developmental trajectories of cell populations during embryonic development.

The SCST methodology can be applied to construct molecular cell atlases with single-cell spatiotemporal resolution for a variety of biospecimens – including embryos, organs, and tissues with stereotypic architecture – from other model organisms. For the gastrula-stage mouse embryo, a geometric model embedded with spatial positional references was devised to recapitulate the spatial architecture of the embryo for cell mapping. In this context, the application of the SCST method to other biospecimens requires the construction of a geometric model that captures the architecture of the biospecimen. This model incorporates a universally valid positional register, which thereby enables the integration of relevant pre-existing scRNA-seq datasets to build a comprehensive molecular single-cell atlas. Meanwhile, through the integration of spatial transcriptomics with single-cell multi-omics technologies such as CITE-seq^26^ and SNARE-seq,^27^ SCST will enable the visualization of true 3D spatial multi-omics information at single-cell resolution. Given the multiple attributes of the SCST mapping methodology, this would be a powerful tool for future research in single-cell spatial transcriptomics and further at the multi-omics level.

### Limitations of the study

A limitation of the SCST methodology is that it is not a fully automated approach and requires parameter adjustment at each step. MDSC Mapping shows a higher level of accuracy compared with other spatial mapping methods, but it requires the prior identification of spatially variable genes. The 3D modeling step involves manual construction of the model by researchers, guided by the target tissue’s anatomy. The Gradient Sort algorithm relies on known gradient-expressed genes or signaling activities. We are directing our efforts toward increasing the automation of the SCST methodology. In this study, we applied SCST to mouse and zebrafish embryos, and we will extend its application to a wider range of biological research scenarios in future studies.

## Resource availability

### Lead contact

Requests for further information and resources should be directed to and will be fulfilled by the lead contact, Naihe Jing (njing@sibcb.ac.cn).

### Materials availability

This study did not generate new unique reagents.

### Data and code availability

The RNA-seq data generated in this study were deposited in the NCBI Gene Expression Omnibus under accession number GSE171588. The scRNA-seq data of single cells sampled from known positions in embryos can be downloaded from “scGastrulation” at http://scgastrulation.sibcb.ac.cn. For the 10× Genomics data, raw and processed single-cell data can be downloaded following the instructions at https://github.com/MarioniLab/EmbryoTimecourse2018. All other data are available from the corresponding authors upon request.

We established a web portal – “scGastrulation” at http://scgastrulation.sibcb.ac.cn to provide the space-, time-, and single-cell resolved transcriptome data as a research resource for the scientific community. The original code of this study is available at “scGastrulation.” We also deposited the original code of this study in a GitHub repository https://github.com/WangRanLab/SCST.

Any additional information required to reanalyze the data reported in this article is available from the lead contact upon request.

## Acknowledgments

This work was supported in part by the National Key Basic Research and Development Program of China (2019YFA0801402, 2018YFA0107200, 2018YFA0801402, 2018YFA0800100, 2018YFA0108000, and 2017YFA0102700), the Strategic Priority Research Program of the Chinese Academy of Sciences (XDA16020501 and XDA16020404), 10.13039/501100001809National Natural Science Foundation of China (31630043, 31900573, 31900454, 31871456, 32130030, and 32570955), the Young Talent Project of 10.13039/100031935Xi'an Jiaotong University (71211224020701), and 10.13039/501100002858China Postdoctoral Science Foundation Grant (2018M642106). P.P.L.T. was supported by the 10.13039/501100000925National Health and Medical Research Council of Australia (Research Fellowship grant 1110751).

## Author contributions

N.J., J.C.M., P.P.L.T., and R.W. conceived the study. X.Y. and Y.Q. generated the experimental results. R.W., C.L., J.W., J.C., and L.W. analyzed the sequencing data. R.W., L.W., and J.C.M. generated the mathematical models. R.W. and Y.X. designed the website. R.W., P.P.L.T., and N.J. wrote the article with the help of all other authors.

## Declaration of interests

The authors declare no competing interests.

## STAR★Methods

### Key resources table

**Table:** 

REAGENT or RESOURCE	SOURCE	IDENTIFIER
**Experimental models: Organisms/strains**		

Mouse: C57BL/6J	Shanghai SLAC laboratory animal company	www.slaccas.com

**Critical commercial assays**		

Multiplex Fluorescent Assay v2	ACDBio	Cat No. 323100

**Chemicals, peptides, and recombinant proteins**		

Tissue-TekO.C.T. Compound	Sakura	4583
RNAscope® Probe- Mm-Mesp1-C3	ACDBio	Cat No. 436281-C3
RNAscope® Probe- Mm-Pou3f1-C2	ACDBio	Cat No. 436421-C2
RNAscope® Probe- Mm-T-C3	ACDBio	Cat No. 423511-C3
RNAscope® Probe- Mm-Foxa2-C4	ACDBio	Cat No. 409111-C4

**Other**		

Leica CM1950 Cryostat	Leica Biosystems	RRID: SCR_018061
Carl Zeiss LSM 980	Carl Zeiss	RRID: SCR_025048

**Software and algorithms**		

Matlab	https://www.mathworks.com MathWorks	R2024b RRID: SCR_001622
R	https://www.r-project.org/ The R Project for Statistical Computing	v4.4.1 RRID: SCR_001905
Python	https://www.python.org/ The Python Software	v3.12.0 RRID: SCR_008394
FASTQC	https://www.bioinformatics.babraham.ac.uk/projects/fastqc/ Babraham Bioinformatics	v0.11.8 RRID: SCR_014583
HISAT2	https://daehwankimlab.github.io/hisat2/ A Python tool for sequencing reads alignment	v2.2.0 RRID: SCR_015530
Seurat	https://satijalab.org/seurat/ An R package for single-cell data analysis	v4.3.0 RRID: SCR_016341
fastMNN	https://www.ebi.ac.uk/research/marioni/software/ An R package for single-cell data analysis	v1.8.0 RRID: SCR_017351
CytoSPACE	https://github.com/digitalcytometry/cytospace A Python package for spatial mapping of single cells	v1.1.0 RRID: SCR_027634
Tangram	https://github.com/broadinstitute/Tangram A Python package for spatial mapping of single cells	v1.0.4 RRID: SCR_006152
novoSpaRc	https://github.com/rajewsky-lab/novosparc A Python package for spatial reconstruction of single-cell data	v0.4.3 RRID: SCR_027635
CSOmap	https://github.com/zhongguojie1998/CSOmap An R package for spatial reconstruction of single-cell data	v1.0 RRID: SCR_027636

### Experimental model and study participant details

The C57/BL6J mice used in this study were purchased from Shanghai SLAC Laboratory Animal Co., Ltd. (www.slaccas.com). All animal experiments were performed at the Animal Core Facility, under program project SIBCB-S308-1807-025 approved by the Institutional Animal Care and Use Committee of the CAS Center for Excellence in Molecular Cell Science, Shanghai Institute of Biochemistry and Cell Biology, Chinese Academy of Sciences. No sex information was collected in this study.

In this study, E6.75 and E7.0 mouse embryos were collected for RNAscope validation. Embryos at each developmental timepoint of gastrulation were staged based on the proximal-distal span of the primitive streak and the anterior-posterior span of the mesoderm layer.^28^ For each stage, 2-3 pregnant mice were sacrificed to collect the embryos. On average, 6-8 embryos were collected from each dam. The expression of *Pou3f1*/*T*/*Foxa2* in E6.75 and *Mesp1* in E7.0 mouse embryos was validated by RNAscope (see the detailed procedure of the RNAscope experiment in the section method details - RNAscope).

### Method details

#### 3D modeling for embryo structure and visualization of Geo-seq data

To mimic the spatial structure and visualize the spatial pattern of gene expression in the embryo, 1) we first developed a geometric concentric half-oval model (formula-i) that can recapitulate the architecture of the cup-shaped gastrula-stage mouse embryo.

(Equation 1) $$\left\{ \begin{array}{c} x^{2}+y^{2}+\frac{z^{2}}{{\left(L_{P-D}/L_{A-P}\right)}^{2}}=r^{2}\\ r_{Ect}=\frac{L_{A-P}^{Ect}}{2},r_{Mes}=\frac{L_{A-P}^{Mes}}{2},r_{End}=\frac{L_{A-P}^{End}}{2} \end{array}\right.$$

2) Generation of the 3D Corn Plot model. For example, at E7.5 stage, *L*_*A*-*P*_/*L*_*P*-*D*_ ≈ 1, ${\;L}_{A-P}^{End}$/ $L_{A-P}^{Mes}$ ≈ 1.2, ${\;L}_{A-P}^{Mes}$/ $L_{A-P}^{Ect}$ ≈ 1.2. The parameters of the 3D Corn Plot model were set in proportion to the scale of real embryo (Figure S1B). Then, we set the following parameters (formula-ii) for the mathematical model.

(Equation 2) $$\left\{ \begin{array}{l} L_{P-D}=9\\ L_{A-P}^{End}=9\\ L_{A-P}^{End}/L_{A-P}^{Mes}=1.2\\ L_{A-P}^{Mes}/L_{A-P}^{Ect}=1.2 \end{array}\right.$$

Applying this model, each Geo-seq position was given a spatial coordinate. For example, the spatial coordinates of the positions at the 9th section of E7.5 embryo are: 9A (-3, 0, 9), 9P (-3, 0, 9), 9L1 (-3⋅cos(π/3), -3⋅sin(π/3), 9), 9R1 (-3⋅cos(π/3), 3⋅sin(π/3), 9), 9L2 (3⋅cos(π/3), -3⋅sin(π/3), 9), 9R2 (3⋅cos(π/3), 3⋅sin(π/3), 9), 9MA (-3.75, 0, 9), 9MP (3.75, 0, 9), 9EA (-4.5, 0, 9), 9EP (4.5, 0, 9).

(Variables: r, radius; L, length; P-D, the proximal-distal axis; A-P, the anterior-posterior axis; Ect, ectoderm; Mes, mesoderm; End, endoderm. For example, ${\;L}_{A-P}^{Ect}$ represents the length of the anterior-posterior axis of the half-oval model of the ectoderm layer. Geo-seq sampling positions: epiblast/ectoderm – A, anterior; P, posterior; L, left lateral; R, right lateral; L1/R1, left/right anterior lateral, L2/R2, left/right posterior lateral; M, mesoderm – MA, anterior mesoderm; MP, posterior mesoderm; E, endoderm – EA, anterior endoderm; EP, posterior endoderm).

3) The RNA-seq data of cell samples were assigned to the positions defined by spatial coordinates on the ‘3D corn plot’ model to depicted the spatial pattern of expression of the gene or gene group of interest, with the expression levels indicated by a color scale computed from the transcript counts in the RNA-seq dataset.

#### Multi-dimension single-cell mapping (MDSC mapping)

Based on the 3D model, a mathematical algorithm was developed for imputing the location of single cells in the germ layers of the mouse embryo. The mathematical operation included: 1) Identification of spatial zipcodes (described in the section ‘identification of the position specific genes (zipcodes)’). 2) Calculate Spearman's rank correlation coefficient (SRCC): The SRCCs between the expression values of the zipcodes of each single cell and all samples of the reference embryo were computed to generate, for example, 81 SRCC values for each single cell against 81 Geo-seq samples of E7.5 embryo, 3) Apply a spatial smoothing algorithm to determine the high-confidence location of each cell. This mapping method contrasts with the previous imputation method that single cells are mapped to the position of the maximum SRCC value.^6^ While the higher SRCC may indicate a strong probability of matching to a position, there were cases where a single cell could match to several adjoining positions. Therefore, for mapping the single cells, the top 3 matching positions with maximum SRCCs were extracted, then applied the formula-iii below to calculate a 3D coordinate *P*_0_(*x*_0_,*y*_0_,*z*_0_):

(Equation 3) $$f\left(x,y,z\right)=\sum_{i=1}^{3}SRCC_{i}\times \sqrt{{\left(x-x_{i}\right)}^{2}+{\left(y-y_{i}\right)}^{2}+{\left(z-z_{i}\right)}^{2}}$$

((*x*_*i*_,*y*_*i*_,*z*_*i*_)|*i*=1,2,3 represent the spatial coordinates of the top 3 matching positions (Geo-seq positions) for each single cell with maximum SRCCs.).

The distance between this location (*P*_0_) and every Geo-seq sample position (*P*_*j*_) was then calculated, the sample with minimum distance (the pseudocode below) was determined as the best mapped position of the cell.

$$d_{\min}=\min_{j\in \left\{1,2,\ldots \right\}}d\left(P_{0},P_{j}\right)$$

#### Updates of the version 2 of MDSC mapping

We improve the mapping accuracy and operation efficiency in the version 2 of MDSC Mapping. The details include: 1) In this version, we implement a ‘germ-layer-sensitive’ operation rule. After calculating the SRCCs between each single cell and all samples (spatial positions) of the reference embryo, the spatial position of the maximum SRCC value determines the germ layer information of the single cell, the top 2 and 3 matching positions are then selected within the spatial positions of the settled germ layer. 2) We adjust the spatial-smoothing algorithm to deal with the case that a cell's top 3 best-matching positions are located in different parts of the embryo (for example, the top 3 matching positions are 11P, 7R and 4L in E7.0 embryo). In this case, the top 5 matching positions are extracted followed by applying the spatial smoothing algorithm to determine the best mapped position of the cell. If the distance between the mapped position and the position of the maximum SRCC value are over 3 sections, we take the latter as the best-fit mapping position. 3) The SRCC value is incorporated into the spatial smoothing algorithm as a weight coefficient to enhance the mapping accuracy, and for ΔSRCC (SRCC_top1 – SRCC_top2) / SRCC_top1 > 0.1, which indicates a single cell map to a specific spatial position with high-confidence, we do not apply the spatial smoothing algorithm. 4) We optimize the code of the mapping algorithm using MATLAB 2024b to improve the operation efficiency. 5) We perform experimental validations and comparative analyses to evaluate the precision of positional mapping (see the “quantification and statistical analysis” section).

#### Single cell datasets for positional mapping

The 10X Genomics single cell data were downloaded as raw files from the Gastrulation Atlas: https://github.com/MarioniLab/EmbryoTimecourse2018. Steps of quality control, normalization, batch correction and clustering were performed using the same criteria as previously described.^10^

#### 3D modeling for single-cell resolution map

To visualize the spatial distribution pattern of single cells, an Annulus Model was developed to reconstruct the embryo spatial structure in single-cell resolution. The model comprises 1) Concentric annuli in the anatomical section of the embryo, from the inside outward, representing epiblast/ectoderm, mesoderm and endoderm germ layer respectively. 2) Division of the annulus into interior spaces matching the Geo-seq defined position. For example, formula-iv shows the function of the constraint conditions of the mathematical model for position 8P at E7.5 stage.

(Equation 4) $$\left\{ \begin{array}{l} {r_{1}}^{2}<x^{2}+y^{2}<{r_{2}}^{2}\\ 0<y<\tan\left(\frac{\pi }{3}\right)\cdot x\\ 7.5<z<8.5 \end{array}\right.$$

Single cells that mapped to a Geo-seq position, were distributed uniformly across the interior space of the position in each annulus section. 3) Within each interior space, apply Gradient Sort algorithm to align the cells along the known gradient of gene expression level or signaling intensity. The Gradient Sort algorithm was designed based on Quick Sort algorithm. For example, on the basis that *Bmp4* expression in the posterior epiblast/ectoderm streak decreases along the proximal-distal axis of the embryo,^29^ the Gradient Sort algorithm rearranged cells in domain 9P in the E7.5 ectoderm (the example below shows the reassigned spatial coordinates for each single cell based on the gradient of *Bmp4* expression, z_1_ > z_2_ > z_3_ > z_4_):

$$\begin{array}{ccc} \begin{array}{c} \text{Cell}\\ \text{Cell}-1\\ \begin{array}{c} \text{Cell}-2\\ \begin{array}{c} \text{Cell}-3\\ \text{Cell}-4 \end{array} \end{array} \end{array} & \begin{array}{c} \begin{array}{c} X\\ X_{1}\\ X_{2} \end{array}\\ X_{3}\\ X_{4} \end{array} & \begin{array}{ccc} \begin{array}{c} \begin{array}{c} Y\\ Y_{1} \end{array}\\ X_{2}\\ X_{3} \end{array} & \begin{array}{c} \begin{array}{c} Z\\ Z_{1} \end{array}\\ Z_{2}\\ Z_{3} \end{array} & \begin{array}{c} \begin{array}{c} \text{Bmp}4\\ 0 \end{array}\\ 3\\ 1 \end{array}\\ X_{4} & Z_{4} & 2 \end{array} \end{array}\to \begin{array}{ccc} \begin{array}{c} \text{Cell}\\ \text{Cell}-2\\ \begin{array}{c} \text{Cell}-4\\ \begin{array}{c} \text{Cell}-3\\ \text{Cell}-1 \end{array} \end{array} \end{array} & \begin{array}{c} \begin{array}{c} X\\ X_{1}\\ X_{2} \end{array}\\ X_{3}\\ X_{4} \end{array} & \begin{array}{ccc} \begin{array}{c} \begin{array}{c} Y\\ Y_{1} \end{array}\\ X_{2}\\ X_{3} \end{array} & \begin{array}{c} \begin{array}{c} Z\\ Z_{1} \end{array}\\ Z_{2}\\ Z_{3} \end{array} & \begin{array}{c} \begin{array}{c} \text{Bmp}4\\ 3 \end{array}\\ 2\\ 1 \end{array}\\ X_{4} & Z_{4} & 0 \end{array} \end{array}$$

This imputation enabled the assignment of spatial coordinate specific for each single cell, and 4) Construct the 3D positional map for all single cells for the visualization of the spatial pattern of the single cells of different cell/tissue lineages that are individually defined by spatial location and transcriptome features. Further information can be drawn from the 3D embryo map for the position of single cells displaying different level of expression (indicated by the color scale computed from the transcript counts in the ‘Gastrulation Atlas’) of gene/gene group of interest.

#### The expanding mesoderm model

To model the mesoderm during gastrulation, we refined the Annulus Model by devising a Gradually Extended Annulus Model for the mesoderm of E7.0 and E7.25 embryos. From distal to proximal, the incomplete annuli that represent the anatomical section of the mesoderm layer that is progressively expanding from posterior to anterior across the embryo. At E7.25, the annuli of proximal mesoderm (7-12M) completely encircles the epiblast. Gradient Sort Algorithm was then applied to assign coordinates to each single cell within the interior space of each annulus of the mesoderm.

#### Nomenclature for cell type annotation

The nomenclature ‘X→Y’ and ‘X(Y)’ represent different cell states. X represents the germ layer information of cell population. ‘X→Y’ indicates these cells are representing a transitional cell state from X to Y. And ‘X(Y)’ represents the precursor of a specified cell type (Y) in the germ layer X.

#### Optimal spatial distribution algorithm

To authentically simulate the spatial distribution pattern of single cells within each Geo-seq position, an Optimal Spatial Distribution algorithm based on Euclidean distance was developed. The mathematical operations include: 1) Input gene set selection. We first cluster the single cells within the Geo-seq position in t-SNE/UMAP space to preview the cellular heterogeneity. Following the Seurat pipeline,^18^ the top PC loading genes are selected as the input gene set. Based on these genes, the gene-expression matrix of single cells within the specific position is prepared and transformed into logarithmic space using log_2_((normalized count)+1). 2) Based on the logarithmic transformed matrix, calculate the Euclidean distance between every two cells to generate the Euclidean distance matrix (*D*_*EDM*_). In order to adapt *D*_*EDM*_ to the interior space of Annulus Model, the *D*_*EDM*_ was normalized through the formula-v below to make the maximum matrix distance (*max*⁡(*d*_*i*,*j*_)) equivalent to the maximum length (*max*⁡(*length*)) within the interior space.

(Equation 5) $$D_{Norm}=D_{EDM}\times \frac{\max\left(length\right)}{\max\left(d_{i,j}\right)}$$

(3) Apply the Least Square Method (vi) under the constraint conditions of the spatial mathematical model of the interior space to derive the optimized coordinate for each single cell:

(Equation 6) $$f=\sum_{i=1}^{n}\sum_{j=1}^{n}{\left(\sqrt{{\left(x_{i}-x_{j}\right)}^{2}+{\left(y_{i}-y_{j}\right)}^{2}+{\left(z_{i}-z_{j}\right)}^{2}}-d_{ij}\right)}^{2}$$

$$\left\{ \begin{array}{l} {r_{1}}^{2}<x^{2}+y^{2}<{r_{2}}^{2}\\ 0<y<\tan\left(\frac{\pi }{3}\right)\cdot x\\ 7.5<z<8.5 \end{array}\right.$$

(This example shows the constraint conditions of the mathematical model for position 8P at E7.5 stage) and 4) Visualize the spatial pattern of single cells of different types in each position. All the spatial algorithms and 3D modelling were operated using MATLAB.

#### Digital lineage tracing algorithm

To trace the developmental trajectory of cell populations in different spatial domains, we developed a Digital Lineage Tracing algorithm with the following computational procedures: For cell populations: 1) Average the gene expression level (log2-transformed) of single cells within each cell population. 2) Use the differentially expressed genes of pair of stages of interest as the input gene set. 3) Calculate the Euclidean distance of any two cell populations from adjacent stages (for example, formula-vii shows the distance between C1_A and C2_B, C1_A denotes the gene expression level of cell population 1 at time point A, C2_B denotes the gene expression level of cell population 2 at time point B).

(Equation 7) $$d\left(C1\_A,C2\_B\right)=\sqrt{\sum_{i=1}^{k}{\left(C1\_A_{i}-C2\_B_{i}\right)}^{2}}$$

4) Calculate the mutual nearest neighbours (minimum distance and variation less than 10%, (*d*_*max*_-*d*_*min*_)/*d*_*min*_<10%) for each cell population from one developmental timepoint against the cell populations from the next developmental timepoint and connect the cell populations. This procedure was repeated across the timepoints from the beginning to the end of the developmental series. 5) Convert the distance of any two connected cell populations to logarithmic space by using log_2_ transformation and visualize the genealogy of cell populations by Sankey plot.

For single cells: To trace the developmental trajectory of individual single cells, after calculating the Euclidean distance of any two single cells from adjacent stages, we only select the minimum distance, and visualize the trajectory in t-SNE/UMAP space.

#### Annotation of cell identity

Based on the single-cell spatial atlas of the gastrulating mouse embryo we established, we annotated the cell identity of the mapped cells to the equivalent *in vivo* cell type. The steps are 1) Extract the differentially expressed genes (DEGs) of each cell type of each Geo-seq position; 2) Calculate Pearson Correlation Coefficients (PCC): The PCC between the expression values of the cell-type DEGs of each mapped cell and all single cells of the inferred position were computed to generate a PCC matrix; 3) Select the maximum PCC value. The single cell at the inferred position that has the maximum PCC value between the mapped cell are identified as the *in vivo* mapping cell. Based on the single-cell spatial atlas, the identity of the mapped cell is then annotated.

#### RNAscope

In this study, E6.75 and E7.0 mouse embryos were collected for RNAscope validation. Gene expression validations to detect *Pou3f1*/*T*/*Foxa2* expression in E6.75 mouse embryo and *Mesp1* expression in E7.0 embryo were performed using RNAscope® Multiplex Fluorescent Reagent Kit v2 (Advanced Cell Diagnostics, 323100) following manufacturer’s instructions. Specifically, E6.75/E7.0 mouse embryos were embedded in OCT matrix and then cryo-sectioned at 15 μm thickness and mounted on electrostatic glass slides. Fresh-prepared slides were fixed using 4% paraformaldehyde solution at room temperature for 30 minutes and then subjected to pretreatment of target retrieval and protease digestion. Specific probes purchased from Advanced Cell Diagnostics (Mm-Pou3f1 (Cat No. 436421-C2), Mm-T (Cat No. 423511-C3), Mm-Foxa2 (Cat No. 409111-C4) and Mm-Mesp1 (Cat No. 436281-C3)) were pre-warmed and then applied for RNA hybridization at 40°C for 2 hours. After that, sequential signal amplification and fluorescent signal detection were performed. Fluorescent images were acquired with the Carl Zeiss LSM980 system.

### Quantification and statistical analysis

#### Data pre-processing

The Geo-seq data of gastrula mouse embryos were retrieved from the NCBI Gene Expression Omnibus (GSE171588). Sequencing quality of raw sequencing data was evaluated by FASTQC. HiSAT2 program^30^^,^^31^ was used to map raw reads to mm39 version of mouse genome with default settings. Mapping ratio was calculated based on the number of mapped reads and total reads for each sample. All mapped reads were processed by StringTie^31^ to quantify gene expression levels (measured by FPKM, Fragment Per Kilobase per Million mapped reads). Finally, the expression levels were transformed to logarithmic space by using the log2 (FPKM+1).

#### Identification of the position specific genes (zipcodes)

The zipcodes for each developmental stage were identified independently. The detail procedures are as follows: 1) We applied the Bayesian Information Criterion-Super K means (BIC-SKmeans) algorithm to identify an optimum number of clusters that can best capture the variance in the spatial transcriptome data, and we defined these clusters as spatial domains. 2) Then we identified the inter-domain differentially expressed genes (DEGs) with a P value < 0.05 and fold change > 1.5. The top 50 DEGs from each spatial domain in E6.5 and E6.75 embryos were designated as their respective zipcodes.

For E7.0, E7.25 and E7.5 embryos, zipcodes were identified as follows: 1) We calculated the variance for each gene across all samples, selected the top 6,000 most variable genes, and then performed principal component analysis (PCA). 2) The top 50 highest and 50 lowest PC loading genes from the most significant PCs (E7.0, PC1-4; E7.25, PC1-5; E7.5, PC1-5), which showed inter-domain specific expression patterns, were designated as zipcodes for E7.0, E7.25 and E7.5 embryos respectively.

#### Verification of the mapping efficiency

The efficiency of MDSC Mapping pipeline was evaluated by mapping single cells manually isolated from known positions in E6.5, E6.75, E7.0, E7.25 and E7.5 C57BL/6J embryos. First, embryos were dissected from the decidua in 10% FBS-DMEM medium, and cells were isolated from a specific position of the embryo by mouth pipetting guided by microscopy. Altogether, 19 single cells from E6.5, 32 single cells from E6.75, 37 single cells from E7.0, 24 single cells from E7.25 and 6 single cells from E7.5 were subjected to automated Smart-seq2 amplification and library construction with the Agilent Bravo automatic liquid-handling platform.^32^ Data preprocessing encompassed mapping, quality control and normalization using the same criteria previously established.^6^ Based on the transcriptome data of these single cells, their position was mapped to the reference embryo by MDSC Mapping.

To access the accuracy of the mapping methodology, we performed the following analysis: 1) Apply image processing to identify the spatial positions of single cells isolated by pipetting, 2) Apply Gaussian Distribution to mathematically simulate the Confidence Intervals. 3) Calculate the Pearson Correlation Coefficients (PCC) between the Confidence Intervals and MDSC Mapping results. Through this modeling protocol, we showed that the single cells could be mapped at significant fidelity to their site of origin, the PCC of MDSC mapping per stage is 0.74 (E6.5), 0.89 (E6.75), 0.80 (E7.0), 0.76 (E7.25) and 0.97 (E7.5).

For comparison, we also applied the widely used single-cell mapping methodologies for the position-specific single cells of each developmental stage, including CytoSPACE Mapping,^17^ Seurat Mapping^18^ and mutual nearest neighbors (MNN) Mapping,^19^ and calculate the PCC between the Confidence Intervals and these mapping results. The comparative analyses indicated that MDSC Mapping has reached a higher level of accuracy.

In addition, we evaluated the accuracy of the four spatial mapping algorithms (CytoSPACE, Seurat, MNN, and MDSC) with two other metrics - AUROC^33^ and F1 score.^34^ Consistent with the PCC results, these two parameters validated the high fidelity of MDSC Mapping.

#### Statistical analysis for RNAscope experiment

The statistical details of RNAscope experiments can be found in the figure legends. The data are presented as mean ± SEM. Student’s t test (two-tailed) was performed for statistical analysis between two groups. A value of p < 0.05 was considered significant.

## Supplemental information

Document S1. Figures S1–S13

Table S1. Zipcodes of each developmental stage, related to Figure S2
