# Supplementary material for: Constructing the spatiotemporal atlas of single-cell lineage trajectories in stereotypic biological structures
Source: iScience. 2025 Dec 1;29(1):114307. doi: 10.1016/j.isci.2025.114307 (PMC12774768; doi:10.1016/j.isci.2025.114307)
Supplement: Document S1. Figures S1–S13 [file mmc1.pdf]

iScience, Volume *29*

Supplemental information

Constructing the spatiotemporal atlas of single-cell lineage trajectories in stereotypic biological structures

Ran Wang, Xianfa Yang, Chengfei Lian, Jianjie Wang, Jiehui Chen, Yun Qian, Yaochen Xu, Liantang Wang, John C. Marioni, Patrick P.L. Tam, and Naihe Jing

SUPPLEMENTAL INFORMATION

Document S1. Figures S1–S13

Figure S1

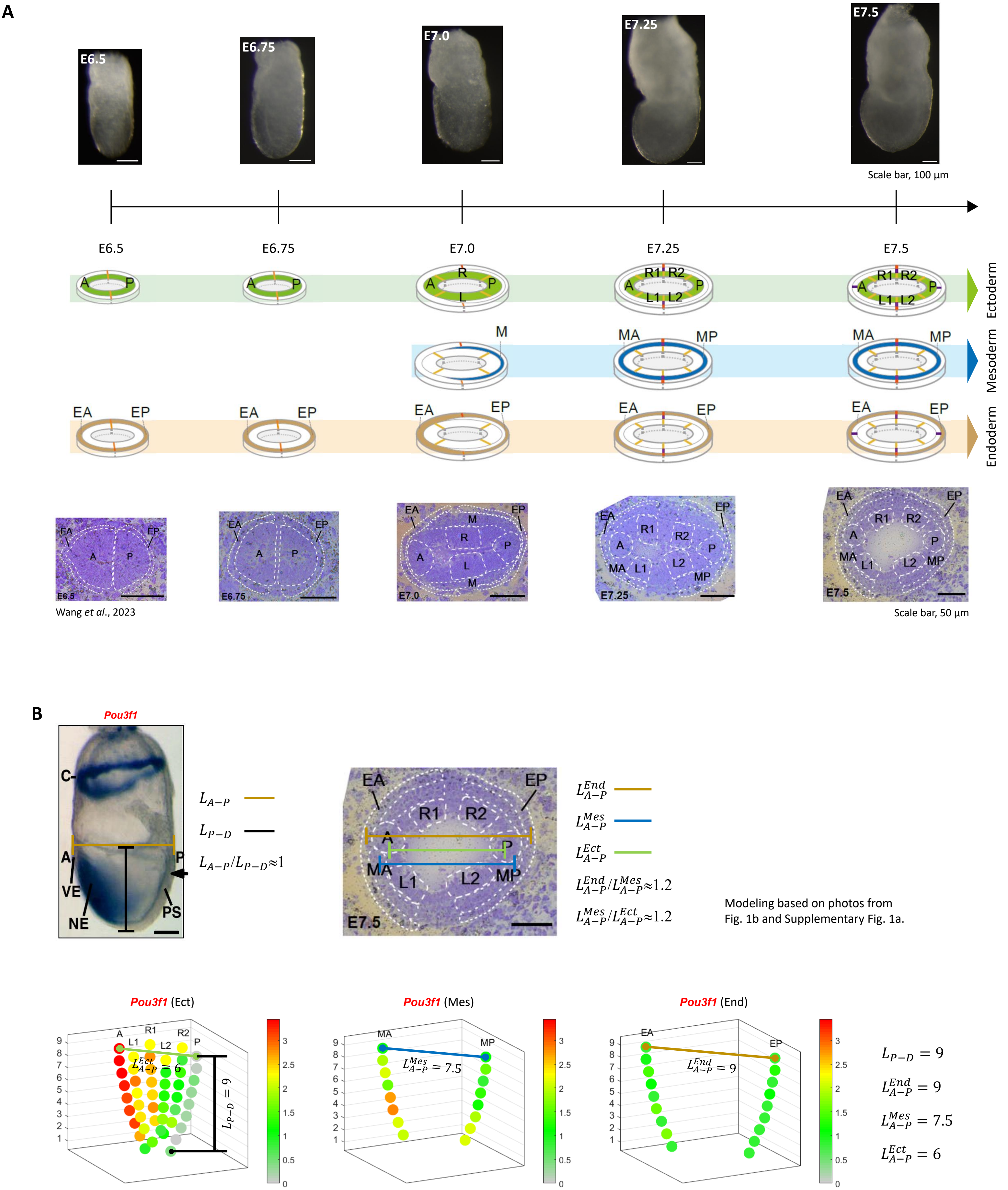
A
E6.5
E6.75
E7.0
E7.25
E7.5
Scale bar, 100 μm
Ectoderm
Mesoderm
Endoderm
A P
R L
R1 R2 L1 L2
M
MA MP
EA EP
Wang et al., 2023
Scale bar, 50 μm
B
Pou3f1
C
A P
VE
NE
PS
L_{A-P}
L_{P-D}
$L_{A-P}/L_{P-D} \approx 1$
L^{End}_{A-P}
L^{Mes}_{A-P}
L^{Ect}_{A-P}
$L^{End}_{A-P}/L^{Mes}_{A-P} \approx 1.2$
$L^{Mes}_{A-P}/L^{Ect}_{A-P} \approx 1.2$
Modeling based on photos from
Fig. 1b and Supplementary Fig. 1a.
Pou3f1 (Ect)
Pou3f1 (Mes)
Pou3f1 (End)
$L^{Ect}_{A-P} = 6$
$L_{P-D} = 9$
$L^{Mes}_{A-P} = 7.5$
$L^{End}_{A-P} = 9$
$L_{P-D} = 9$
$L^{End}_{A-P} = 9$
$L^{Mes}_{A-P} = 7.5$
$L^{Ect}_{A-P} = 6$

Figure S1. Geo-seq analysis and visualization of Geo-seq data. Related to Figure 1.

(A) Schematics of laser capture microdissection (LCM) of cell samples in E6.5-E7.5 embryos (areas of sampling also shown in histology images in the bottom panel, Scale bar, 50 μm). Geo-seq sampling positions: epiblast/ectoderm – A, anterior; P, posterior; L, left lateral; R, right lateral; L1/R1, left/right anterior lateral, L2/R2, left/right posterior lateral; M, mesoderm – MA, anterior mesoderm; MP, posterior mesoderm; E, endoderm – EA, anterior endoderm; EP, posterior endoderm. The image of LCM of cell samples was from previous study by Wang *et al*., 2023.

(B) The parameter settings of the '3D Corn Plot model'. The model was generated based on the actual size of the embryo. Top-left panel: The scale of the length of P-D axis (L_{P-D}) and A-P axis (L_{A-P}) of E7.5 embryo, L_{A-P}/L_{P-D}≈1. Top-right panel: The scale of the diameters of ectoderm, mesoderm and endoderm layer (L_{A-P}^{Ect}, L_{A-P}^{Mes}, L_{A-P}^{End}) of E7.5 embryo. $L_{A-P}^{End}/L_{A-P}^{Mes}$≈1.2, $L_{A-P}^{Mes}/L_{A-P}^{Ect}$≈1.2. Bottom panel: The parameters of the 3D Corn Plot model were set in proportion to the scale of the real embryo.

Figure S2

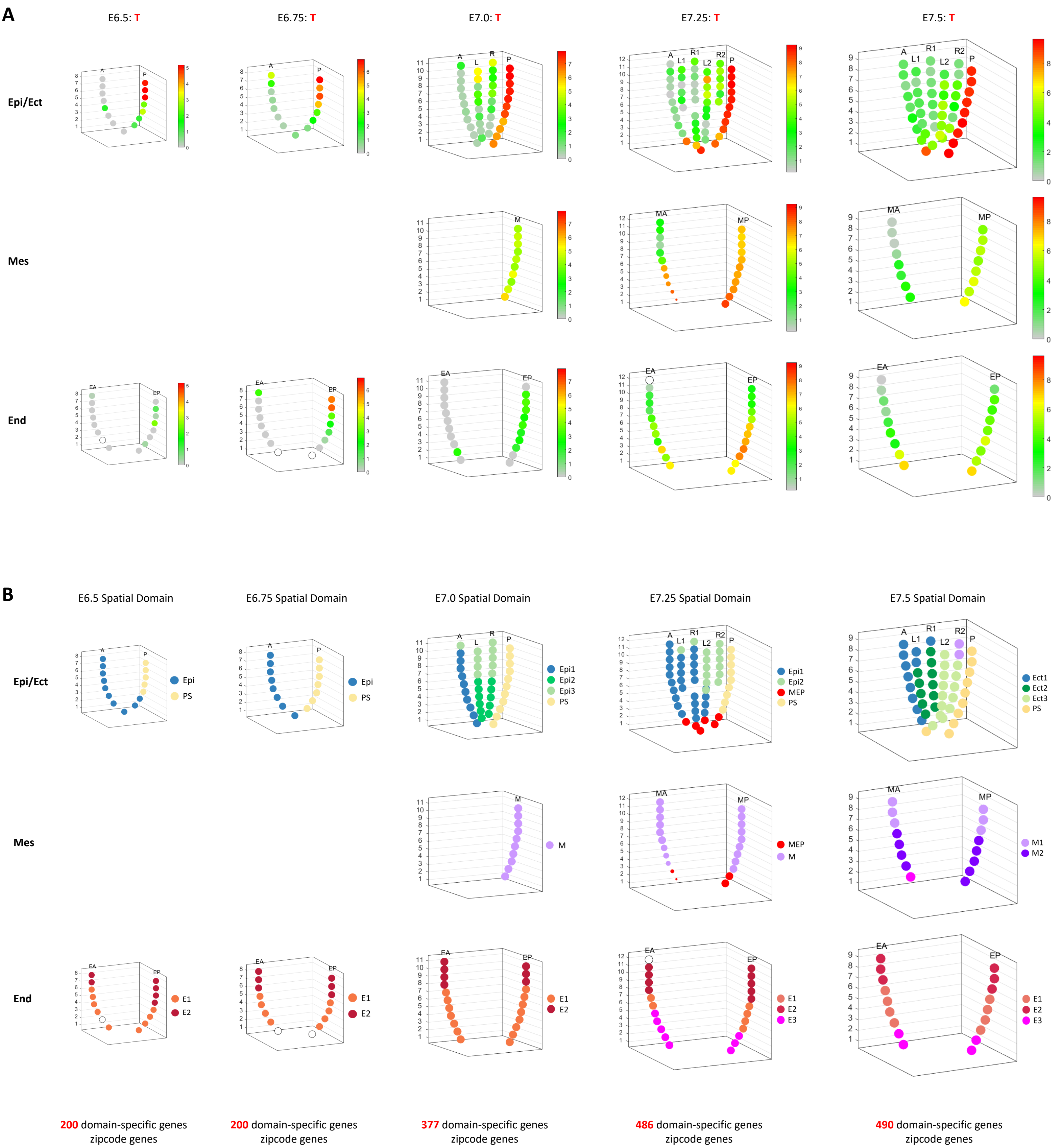

Figure S2. Spatial domain of cell populations in the germ layers of gastrula-stage mouse embryo. Related to Figure 1.

(A) 3D corn plots showing the spatio-temporal distribution of *T*-expressing cells in E6.5-E7.5 embryos. 3D corn plots display the Geo-seq cell samples in epiblast/ectoderm, mesoderm and endoderm layers. The color legend indicates the level of expression determined by the transcript counts.

(B) 3D corn plots showing the spatial domain of cell populations in the epiblast/ectoderm, mesoderm and endoderm of E6.5-E7.5 embryos, defined by the position-specific expression of zipcode genes. Germ layer domains: Epi: epiblast, Epi1, 2, 3: epiblast domain 1, 2 and 3; M: mesoderm, M1, M2: mesoderm domain 1 and 2; MEP, putative mesendoderm progenitors; E: endoderm, E1, E2, E3: endoderm domain 1, 2 and 3; PS, primitive streak.

Figure S3

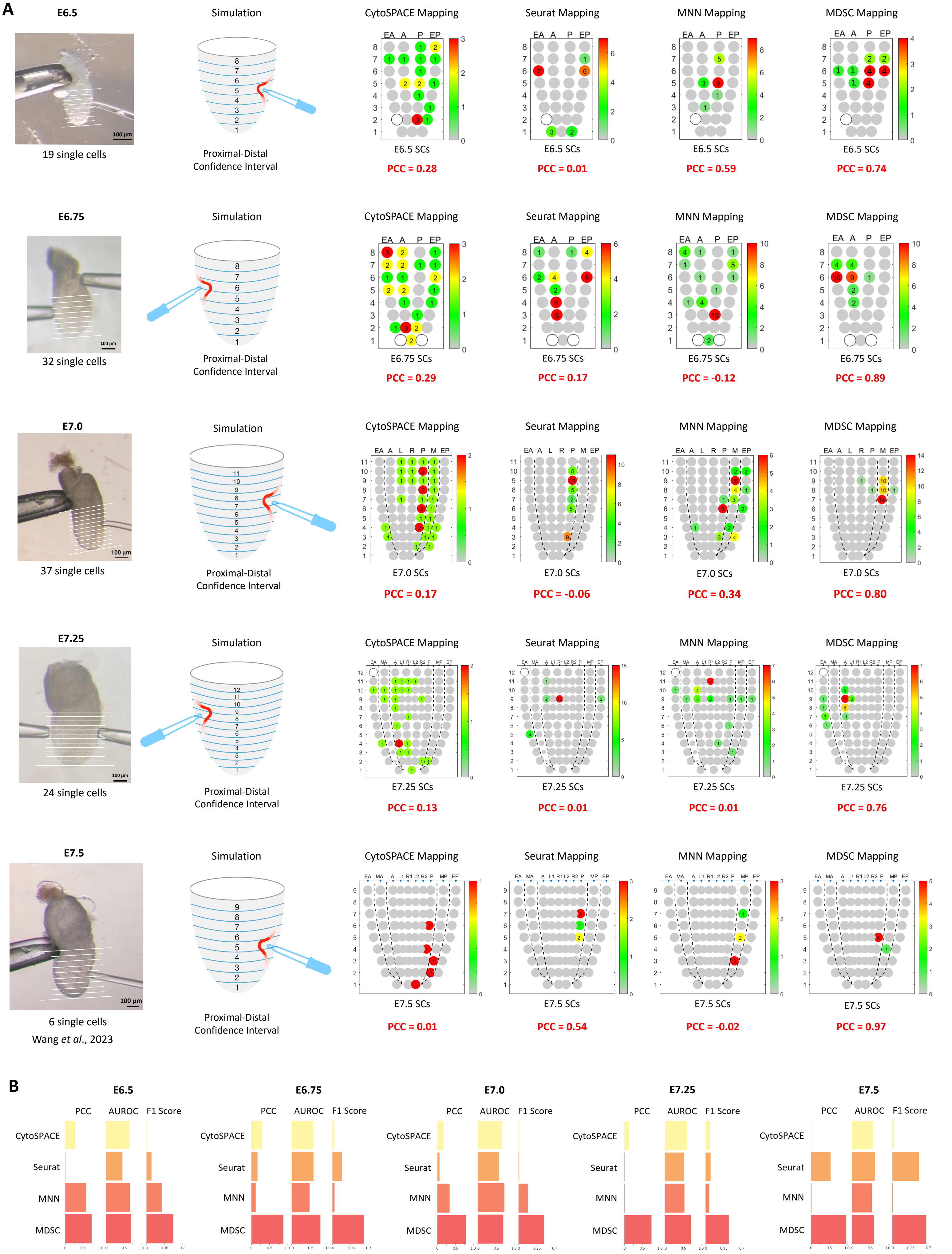
A
E6.5
Simulation
CytoSPACE Mapping
Seurat Mapping
MNN Mapping
MDSC Mapping
19 single cells
Proximal-Distal
Confidence Interval
E6.5 SCs
PCC = 0.28
PCC = 0.01
PCC = 0.59
PCC = 0.74
E6.75
32 single cells
E6.75 SCs
PCC = 0.29
PCC = 0.17
PCC = -0.12
PCC = 0.89
E7.0
37 single cells
E7.0 SCs
PCC = 0.17
PCC = -0.06
PCC = 0.34
PCC = 0.80
E7.25
24 single cells
E7.25 SCs
PCC = 0.13
PCC = 0.01
PCC = 0.01
PCC = 0.76
E7.5
6 single cells
Wang et al., 2023
E7.5 SCs
PCC = 0.01
PCC = 0.54
PCC = -0.02
PCC = 0.97
100 µm
EA A P EP
EA A L R P M EP
EA MA A L1 R1 L2 R2 P MP EP
B
E6.5
E6.75
E7.0
E7.25
E7.5
PCC
AUROC
F1 Score
CytoSPACE
Seurat
MNN
MDSC

Figure S3. Verification of the efficiency of MDSC Mapping. Related to Figure 1.

(A) Benchmarking MDSC Mapping of single cells sampled from known positions of E6.5, E6.75, E7.0, E7.25 and E7.5 embryos (left panels) with CytoSPACE Mapping, Seurat Mapping, mutual nearest neighbors (MNN) Mapping. The number on the corns indicates the number of cells mapped to the specific positions in the germ layers. PCC values and confidence intervals are shown in the simulation. The comparison indicated that MDSC Mapping attained a higher level of accuracy. The image of isolating single cells was obtained from our previous study (Wang *et al*., 2023).

(B) The accuracy of the four spatial mapping algorithms (CytoSPACE, Seurat, MNN, and MDSC) was further assessed with AUROC and F1 score. The evaluation was performed for each developmental stage. The comparison analyses validated the high fidelity of MDSC Mapping.

Figure S4

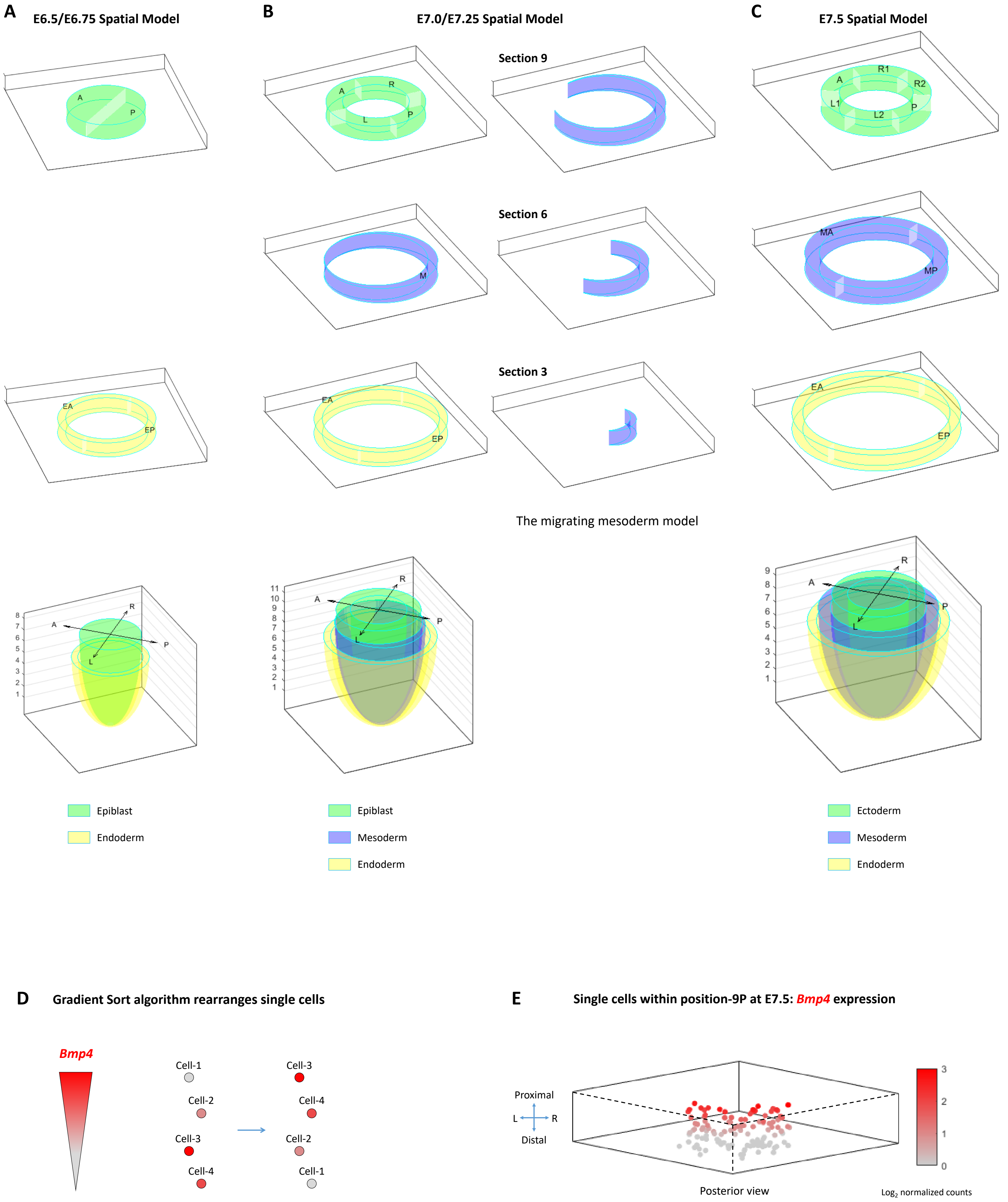

A
E6.5/E6.75 Spatial Model
B
E7.0/E7.25 Spatial Model
C
E7.5 Spatial Model
Section 9
Section 6
Section 3
The migrating mesoderm model
A
P
R
L
M
EA
EP
R1
R2
L1
L2
MA
MP
Epiblast
Endoderm
Epiblast
Mesoderm
Endoderm
Ectoderm
Mesoderm
Endoderm
D
Gradient Sort algorithm rearranges single cells
Bmp4
Cell-1
Cell-2
Cell-3
Cell-4
Cell-3
Cell-4
Cell-2
Cell-1
E
Single cells within position-9P at E7.5: Bmp4 expression
Proximal
L
R
Distal
Posterior view
3
2
1
0
Log2 normalized counts

Figure S4. Geometric models (the Annulus Model) for single-cell resolution mapping. Related to Figure 2.

(A) The 3D model of E6.5 and E6.75 embryos. Based on Geo-seq sampling strategy, semicircular domains for the epiblast and annulus domains for the endoderm are structured for displaying the spatial distribution of single cells.

(B) The annulus model of the epiblast, mesoderm and endoderm of E7.0 and E7.25 embryos. At E7.0-E7.25, the partial annulus domain mirrors the mesoderm layer that is expanding from posterior to anterior of the embryo.

(C) The annulus model of E7.5 embryo. Concentric annuli represent the ectoderm, mesoderm and endoderm from inside outward.

(D) Gradient Sort algorithm for re-ordering single cells in accordance to the gradient of gene expression or signaling activity, e.g., *Bmp4* expression in a proximal to distal descending gradient.

(E) The spatial distribution of *Bmp4*-expressing cells within position-9P of E7.5 embryo. The color legend indicates the level of expression determined by the transcript counts.

Figure S5

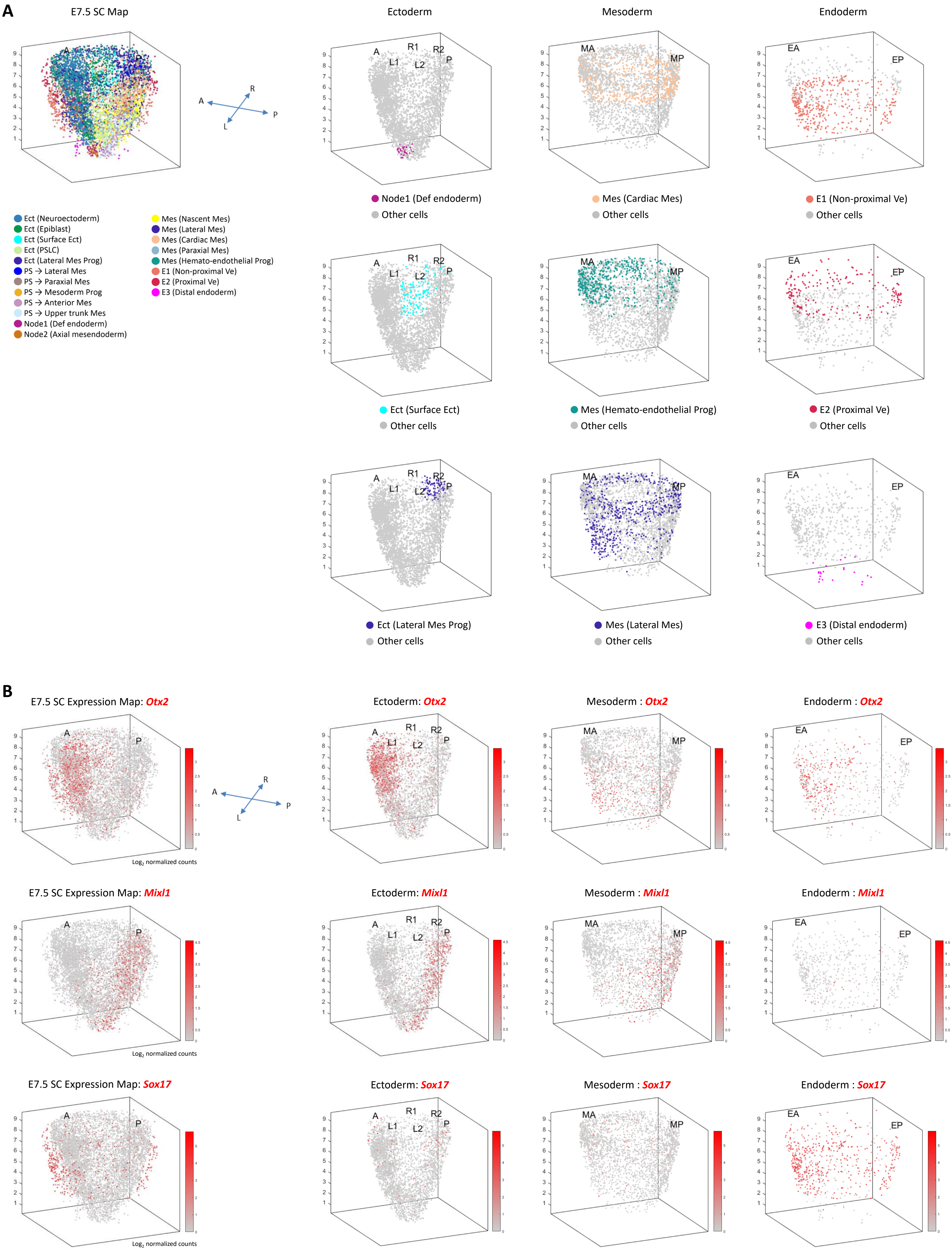
A
E7.5 SC Map
Ectoderm
Mesoderm
Endoderm
A
P
R
L
R1
R2
L1
L2
MA
MP
EA
EP
Ect (Neuroectoderm)
Ect (Epiblast)
Ect (Surface Ect)
Ect (PSLC)
Ect (Lateral Mes Prog)
PS → Lateral Mes
PS → Paraxial Mes
PS → Mesoderm Prog
PS → Anterior Mes
PS → Upper trunk Mes
Node1 (Def endoderm)
Node2 (Axial mesendoderm)
Mes (Nascent Mes)
Mes (Lateral Mes)
Mes (Cardiac Mes)
Mes (Paraxial Mes)
Mes (Hemato-endothelial Prog)
E1 (Non-proximal Ve)
E2 (Proximal Ve)
E3 (Distal endoderm)
Node1 (Def endoderm)
Other cells
Mes (Cardiac Mes)
Other cells
E1 (Non-proximal Ve)
Other cells
Ect (Surface Ect)
Other cells
Mes (Hemato-endothelial Prog)
Other cells
E2 (Proximal Ve)
Other cells
Ect (Lateral Mes Prog)
Other cells
Mes (Lateral Mes)
Other cells
E3 (Distal endoderm)
Other cells
B
E7.5 SC Expression Map: Otx2
Ectoderm: Otx2
Mesoderm : Otx2
Endoderm : Otx2
E7.5 SC Expression Map: Mixl1
Ectoderm: Mixl1
Mesoderm : Mixl1
Endoderm : Mixl1
E7.5 SC Expression Map: Sox17
Ectoderm: Sox17
Mesoderm : Sox17
Endoderm : Sox17
Log₂ normalized counts

Figure S5. The spatial distribution of single cells of E7.5 mouse embryo. Related to Figure 2.

(A) Single-cell (SC) maps showing the spatial distribution of single cells identified in the 'scGastrulation' of E7.5 mouse embryo. Cells are colored by their cell-type annotation (left panel). SC maps of selected cell types are shown in different germ layers (right panel).

(B) SC maps showing the spatial pattern of expression of representative cell markers (*Otx2*, *Mixl1* and *Sox17*) in whole embryos (left panels) and the three germ layers (right panels) at E7.5. The color legend indicates the level of expression determined by the transcript counts.

Figure S6

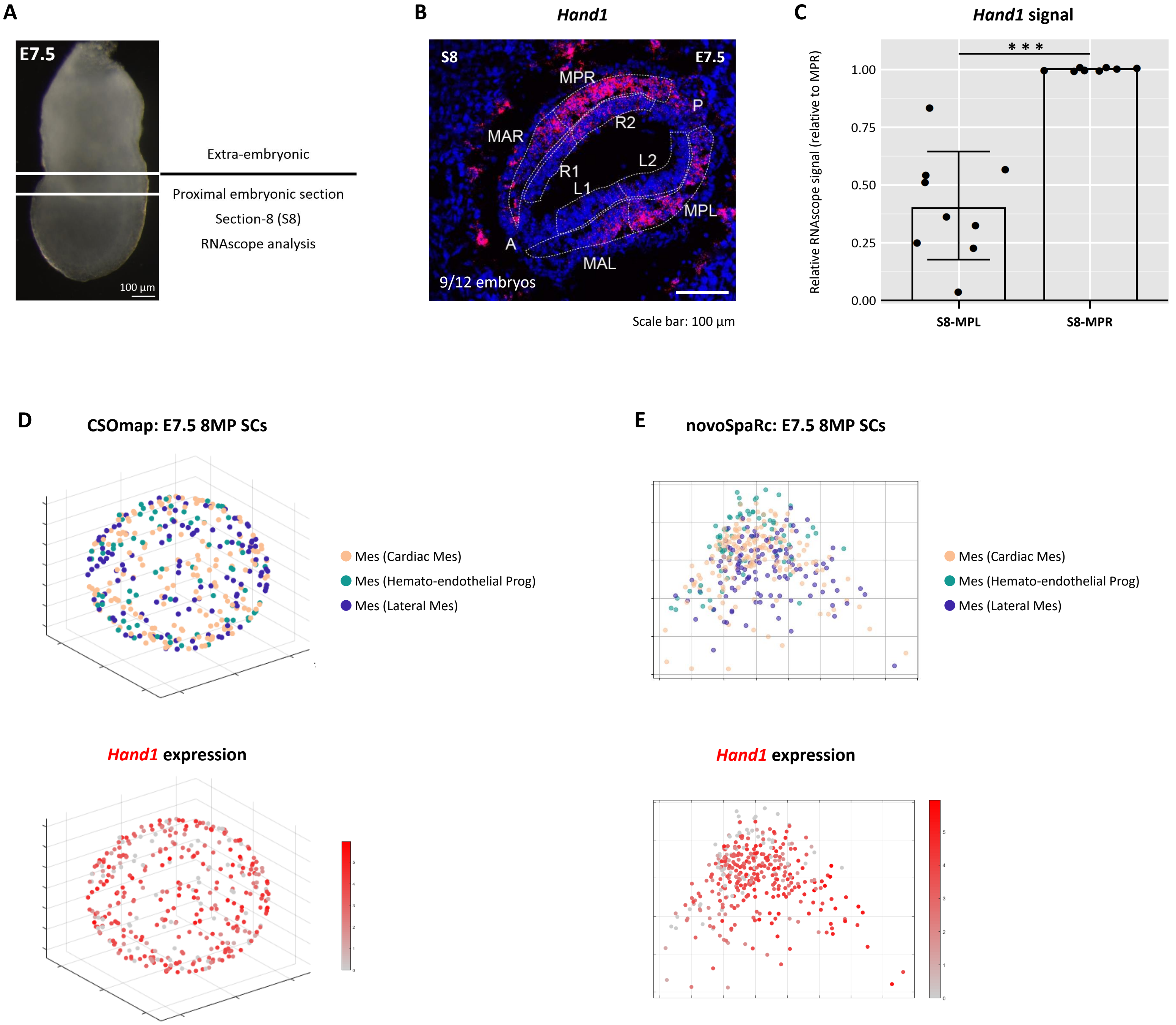

Figure S6. Validation of the pattern of *Hand1* expression. Related to Figure 2.

(A) Section of E7.5 embryo for RNAscope analysis.

(B) RNAscope analysis showing the asymmetric expression of *Hand1* in the mesoderm (n = 12, 9 of the 12 embryos showed asymmetric expression). The mesoderm germ layer was partitioned into different regions: MAL (anterior left mesoderm), MAR (anterior right mesoderm), MPL (posterior left mesoderm) and MPR (posterior right mesoderm).

(C) Scatter plot of the quantified signal intensity. ***, significant difference at $p < 0.001$. The fluorescence intensity of each region (B) was calculated using Image J software following standard steps. In order to minimize signal variance between embryos and experimental batches, the integrated fluorescent intensity of MPL region was normalized by calculating the relative ratio to the MPR region. Data are presented as mean ± SEM. Statistical differences are evaluated with two-tailed unpaired Student's t test.

(D) CSOmap simulates the spatial distribution pattern of single cells of position-8MP of E7.5 embryo (top panel). *Hand1* expression is also shown in the CSOmap simulation (bottom panel).

(E) novoSpaRc simulates the spatial distribution pattern of single cells of position-8MP of E7.5 embryo (top panel). *Hand1* expression is also shown in the novoSpaRc simulation (bottom panel).

Figure S7

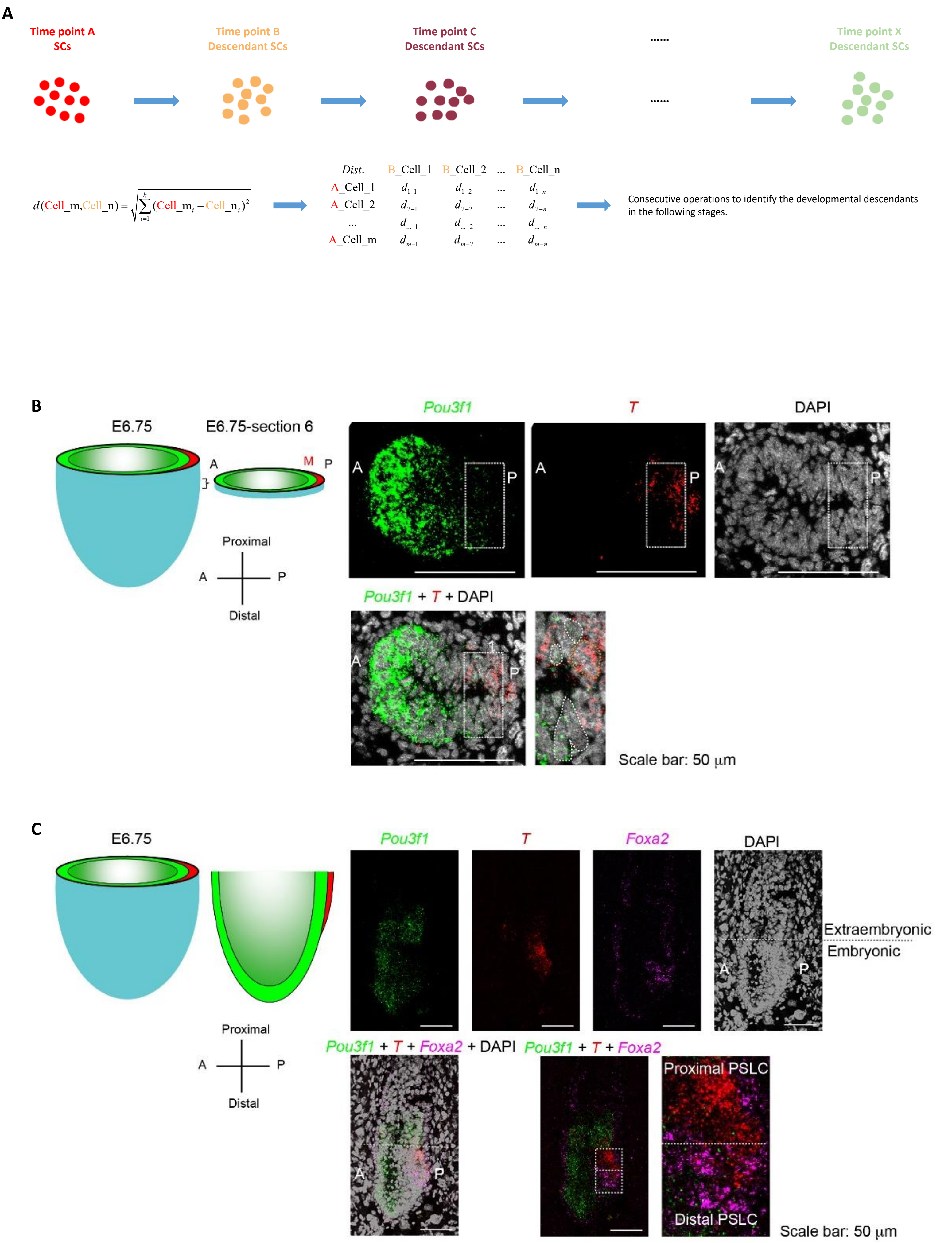
A
Time point A
SCs
Time point B
Descendant SCs
Time point C
Descendant SCs
......
Time point X
Descendant SCs
......
d(Cell_m,Cell_n) = √(Σ_{i=1}^{k} (Cell_m_i − Cell_n_i)^2)
Dist. B_Cell_1 B_Cell_2 ... B_Cell_n
A_Cell_1 d_{1−1} d_{1−2} ... d_{1−n}
A_Cell_2 d_{2−1} d_{2−2} ... d_{2−n}
... d_{...−1} d_{...−2} ... d_{...−n}
A_Cell_m d_{m−1} d_{m−2} ... d_{m−n}
Consecutive operations to identify the developmental descendants in the following stages.
B
E6.75
E6.75-section 6
A
M
P
Proximal
Distal
Pou3f1
T
DAPI
Pou3f1 + T + DAPI
Scale bar: 50 μm
C
E6.75
Pou3f1
T
Foxa2
DAPI
Extraembryonic
Embryonic
Proximal
Distal
Pou3f1 + T + Foxa2 + DAPI
Pou3f1 + T + Foxa2
Proximal PSLC
Distal PSLC
Scale bar: 50 μm

Figure S7. The Digital Lineage Tracing algorithm and the validation of cell type-specific markers. Related to Figure 4.

(A) Schematics of Digital Lineage Tracing algorithm for single cells of E6.5-E7.5 embryos.

(B) RNAscope analyses validated the *Pou3f1*-expressing ('Epi→Ect' lineage) and *T*-expressing cells ('PS→Mes' lineage) in the proximal posterior epiblast of the E6.75 mouse embryo.

(C) RNAscope analyses validated the *Foxa2*-expressing cells ('PSLC', mesendoderm lineage) in the distal posterior epiblast of the E6.75 mouse embryo.

For each RNAscope experimental validation (*Pou3f1*/*T*/*Foxa2*), three biological replicates were examined.

Figure S8

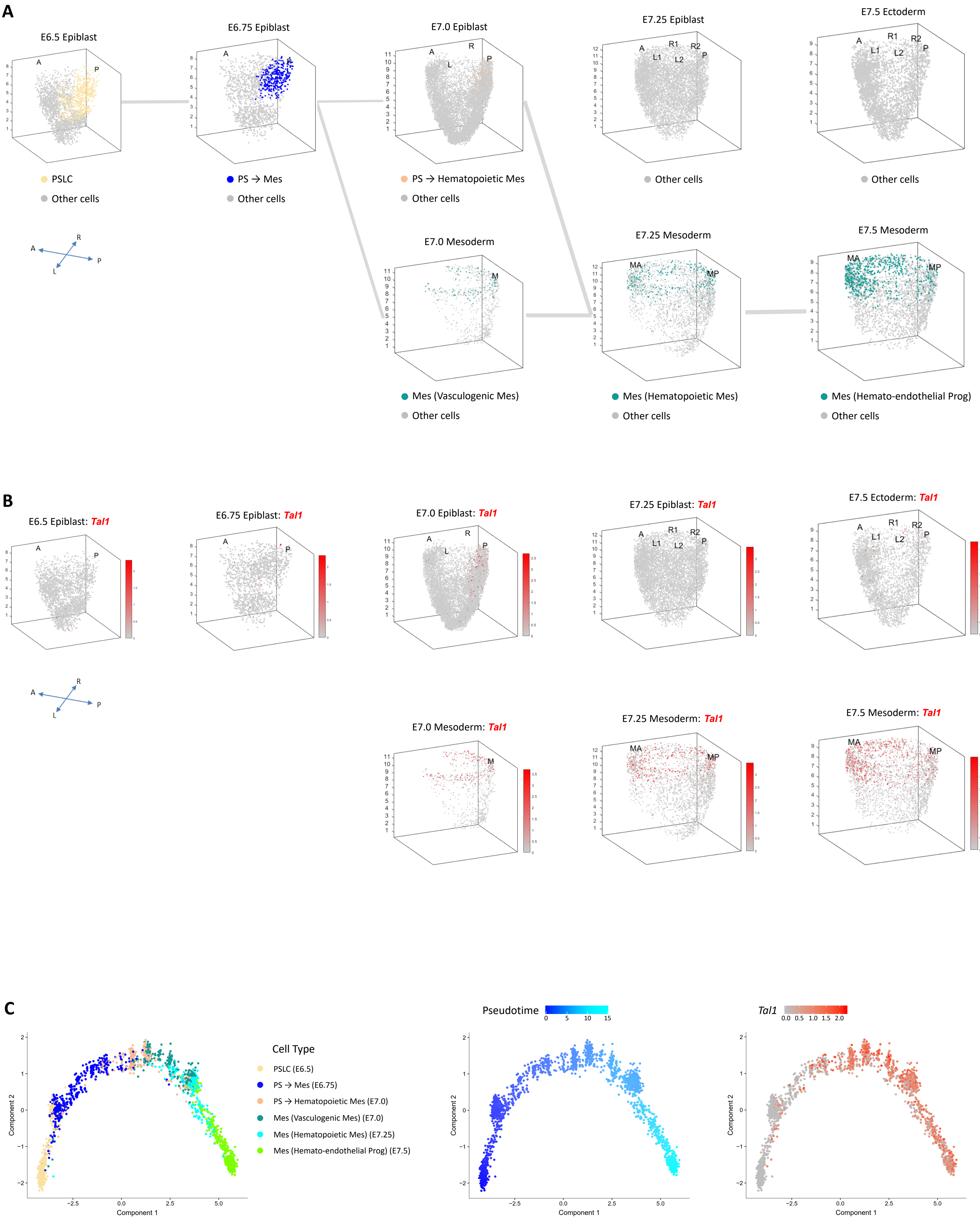

A
E6.5 Epiblast
PSLC
Other cells
E6.75 Epiblast
PS → Mes
Other cells
E7.0 Epiblast
PS → Hematopoietic Mes
Other cells
E7.25 Epiblast
Other cells
E7.5 Ectoderm
Other cells
E7.0 Mesoderm
Mes (Vasculogenic Mes)
Other cells
E7.25 Mesoderm
Mes (Hematopoietic Mes)
Other cells
E7.5 Mesoderm
Mes (Hemato-endothelial Prog)
Other cells
A
R
L
P
B
E6.5 Epiblast: *Tal1*
E6.75 Epiblast: *Tal1*
E7.0 Epiblast: *Tal1*
E7.25 Epiblast: *Tal1*
E7.5 Ectoderm: *Tal1*
E7.0 Mesoderm: *Tal1*
E7.25 Mesoderm: *Tal1*
E7.5 Mesoderm: *Tal1*
C
Cell Type
PSLC (E6.5)
PS → Mes (E6.75)
PS → Hematopoietic Mes (E7.0)
Mes (Vasculogenic Mes) (E7.0)
Mes (Hematopoietic Mes) (E7.25)
Mes (Hemato-endothelial Prog) (E7.5)
Pseudotime
Tal1
Component 1
Component 2

Figure S8. The spatio-temporal developmental trajectory of hematopoiesis lineage. Related to Figure 4.

(A) The spatio-temporal developmental trajectory of hematopoiesis lineage in E6.5-E7.5 embryos.

(B) The spatio-temporal distribution of *Tal1*-expressing cells in E6.5-E7.5 embryos. The color legend indicates the level of expression determined by the transcript counts.

(C) Pseudotime analysis (Monocle) validates the developmental trajectory of hematopoietic lineage imputed using the Digital Lineage Tracing algorithm. Cell types are annotated on the Monocle trajectory in the left panel. Pseudotime labels are assigned to the hematopoietic lineage in the middle panel. Expression pattern of *Tal1* in the hematopoietic lineage is shown in the right panel.

A E7.0 37 single cells

Cell	Position	Cell Type
E7.0_P_1	8EP	E1 (Non-proximal Ve)
E7.0_P_2	9R	Epi → SEct/AmEct
E7.0_P_3	8P	PS → Lateral Mes
E7.0_P_4	8M	
E7.0_P_5	8M	
E7.0_P_6	8M	
E7.0_P_7	8M	
E7.0_P_8	8M	
E7.0_P_9	8M	Mes (Cardiogenic Mes)
E7.0_P_10	9M	
E7.0_P_11	9M	
E7.0_P_12	9M	
E7.0_P_13	9M	
E7.0_P_14	9M	
E7.0_P_15	7M	
E7.0_P_16	7M	
E7.0_P_17	7M	
E7.0_P_18	7M	
E7.0_P_19	7M	
E7.0_P_20	7M	
E7.0_P_21	7M	
E7.0_P_22	7M	
E7.0_P_23	7M	
E7.0_P_24	7M	
E7.0_P_25	7M	
E7.0_P_26	7M	Mes (Paraxial Mes)
E7.0_P_27	7M	
E7.0_P_28	7M	
E7.0_P_29	8M	
E7.0_P_30	8M	
E7.0_P_31	8M	
E7.0_P_32	8M	
E7.0_P_33	9M	
E7.0_P_34	9M	
E7.0_P_35	9M	
E7.0_P_36	9M	
E7.0_P_37	9M	

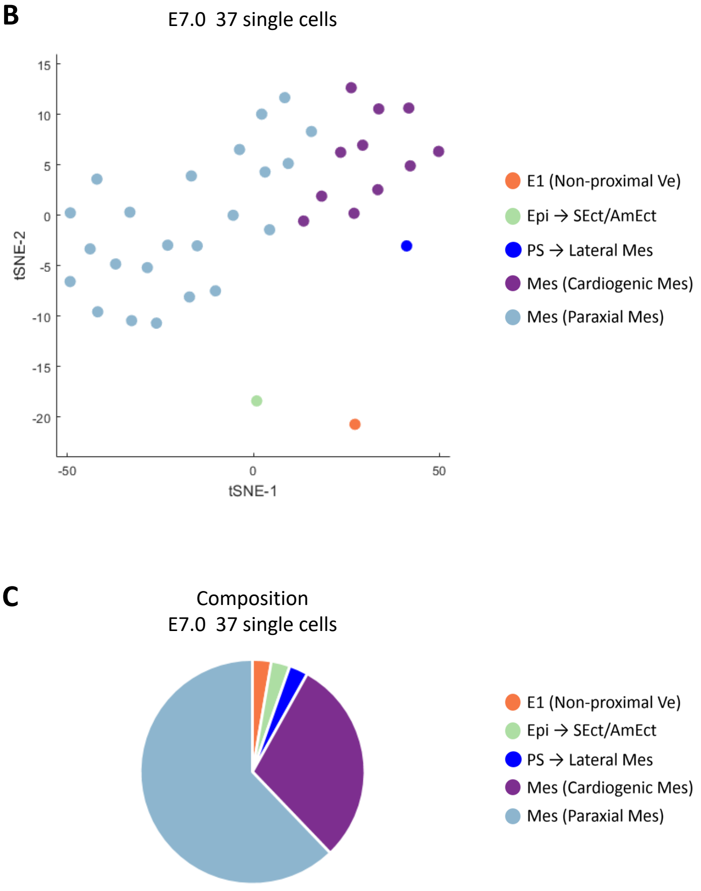

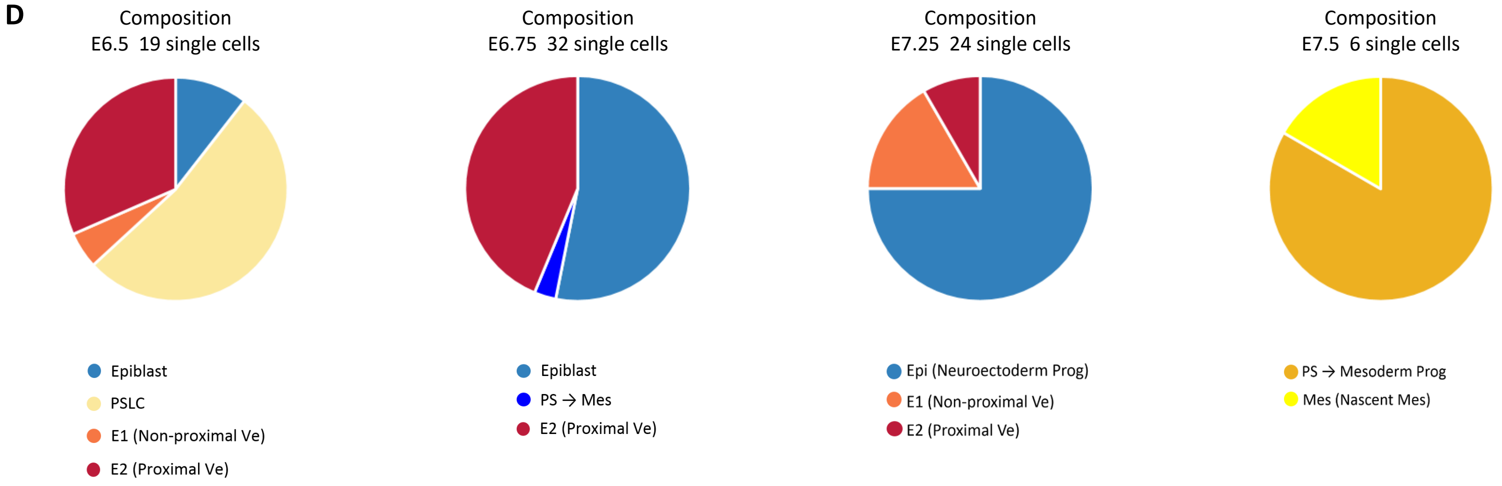

Figure S9. Annotation of cell identity. Related to Figure 5.

(A) MDSC mapping and annotation results for the single cells isolated from the known position in E7.0 mouse embryo.

(B) *t*-SNE plot showing the annotation results for the single cells. Cell types are indicated in the color legend.

(C) Pie chart showing the fraction of single-cell types.

(D) Pie charts showing the fraction of cell types of single cells isolated from other known positions of E6.5, E6.75, E7.25 and E7.5 embryos.

Figure S10

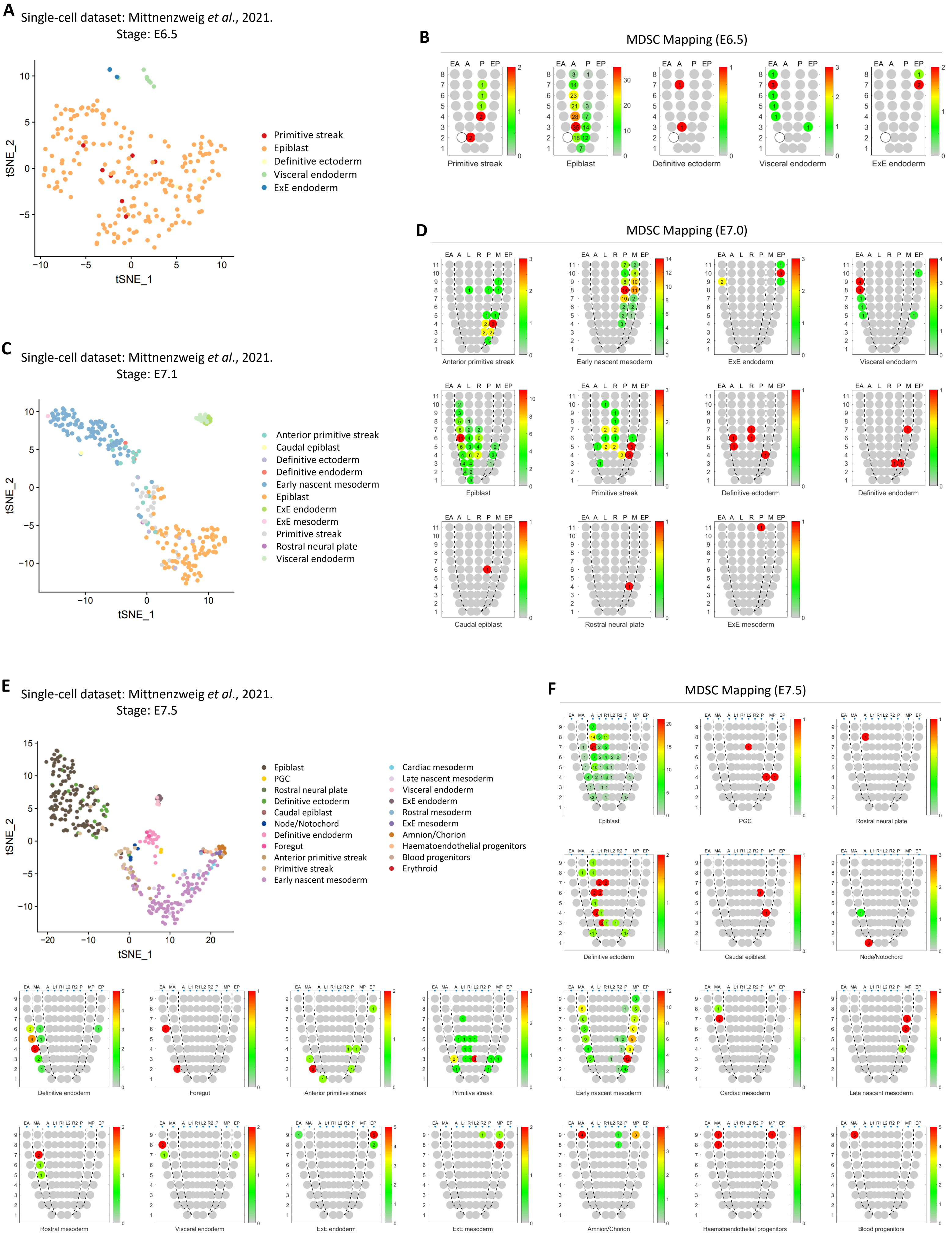
A
Single-cell dataset: Mittnenzweig et al., 2021.
Stage: E6.5
tSNE_1
tSNE_2
Primitive streak
Epiblast
Definitive ectoderm
Visceral endoderm
ExE endoderm
B
MDSC Mapping (E6.5)
EA A P EP
Primitive streak
Epiblast
Definitive ectoderm
Visceral endoderm
ExE endoderm
C
Single-cell dataset: Mittnenzweig et al., 2021.
Stage: E7.1
Anterior primitive streak
Caudal epiblast
Definitive ectoderm
Definitive endoderm
Early nascent mesoderm
Epiblast
ExE endoderm
ExE mesoderm
Primitive streak
Rostral neural plate
Visceral endoderm
D
MDSC Mapping (E7.0)
EA A L R P M EP
Anterior primitive streak
Early nascent mesoderm
ExE endoderm
Visceral endoderm
Epiblast
Primitive streak
Definitive ectoderm
Definitive endoderm
Caudal epiblast
Rostral neural plate
ExE mesoderm
E
Single-cell dataset: Mittnenzweig et al., 2021.
Stage: E7.5
Epiblast
PGC
Rostral neural plate
Definitive ectoderm
Caudal epiblast
Node/Notochord
Definitive endoderm
Foregut
Anterior primitive streak
Primitive streak
Early nascent mesoderm
Cardiac mesoderm
Late nascent mesoderm
Visceral endoderm
ExE endoderm
Rostral mesoderm
ExE mesoderm
Amnion/Chorion
Haematoendothelial progenitors
Blood progenitors
Erythroid
F
MDSC Mapping (E7.5)
EA MA A L1 R1 L2 R2 P MP EP
Epiblast
PGC
Rostral neural plate
Definitive ectoderm
Caudal epiblast
Node/Notochord
Definitive endoderm
Foregut
Anterior primitive streak
Primitive streak
Early nascent mesoderm
Cardiac mesoderm
Late nascent mesoderm
Rostral mesoderm
Visceral endoderm
ExE endoderm
ExE mesoderm
Amnion/Chorion
Haematoendothelial progenitors
Blood progenitors

Figure S10. Mapping the spatial distribution of single cells. Related to Figure 5.

(A-F) *t*-SNE plots (A, C, E) showing the data structure of single cells collated in a published dataset (Mittnenzweig *et al*., 2021) and MDSC Mapping results (B, D, F) for E6.5 (A, B), E7.1 (C, D) and E7.5 (E, F) embryos. Legends of *t*-SNE plots identified the cell types and the spatial distribution of each cell type is displayed in corn plots, with the number of cells mapped to specific Geo-seq position indicated by numbers on the corn.

Figure S11

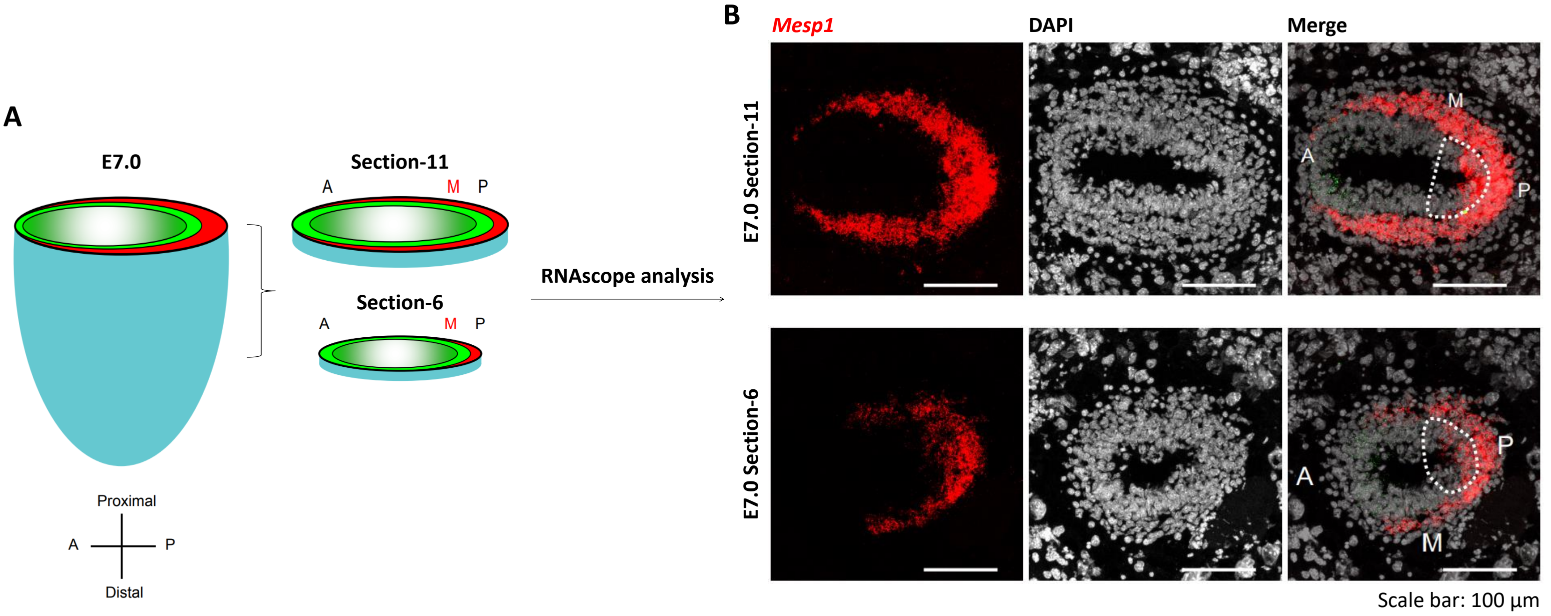

Figure S11. *Mesp1* expression in mouse E7.0 embryo. Related to Figure 5.

(A) Sections of E7.0 embryo for RNAscope analysis.

(B) RNAscope analyses revealed the expression of *Mesp1* in the epiblast E7.0 embryo. *Mesp1*-expressing cells in the posterior epiblast are indicated (dotted circle).

For the RNAscope validation of *Mesp1* expression, three biological replicates were examined.

Figure S12

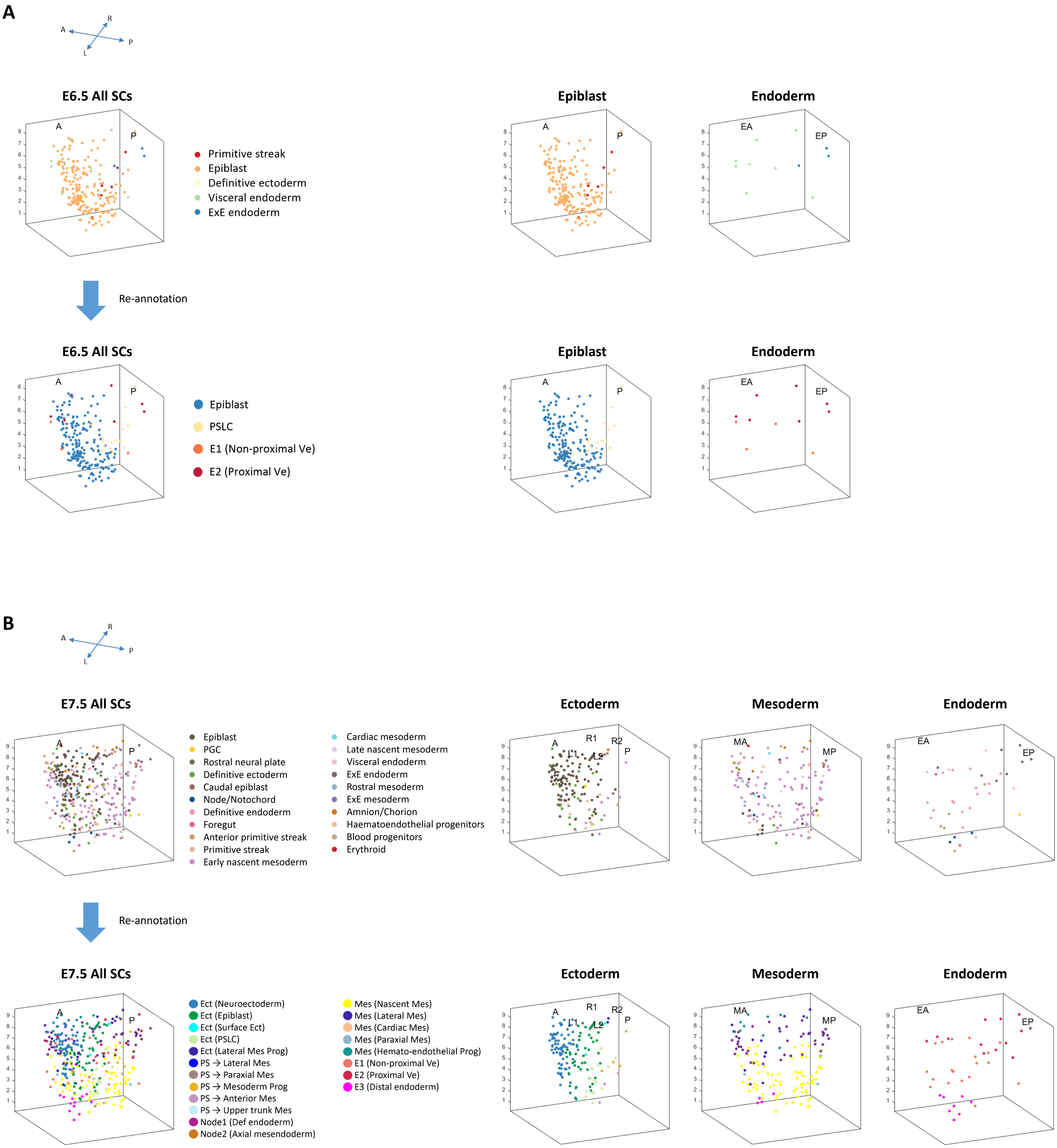

Figure S12. Annotation of cell identity and 3D reconstruction of the single-cell resolved spatial molecular map. Related to Figure 5. (A and B) The spatial distribution of single cells with original cell identity (uppers panels) and re-annotated cell identity (lower panels) in whole embryos (left panels) and in different germ layers of E6.5 (A) and E7.5 (B) embryos (right panels).

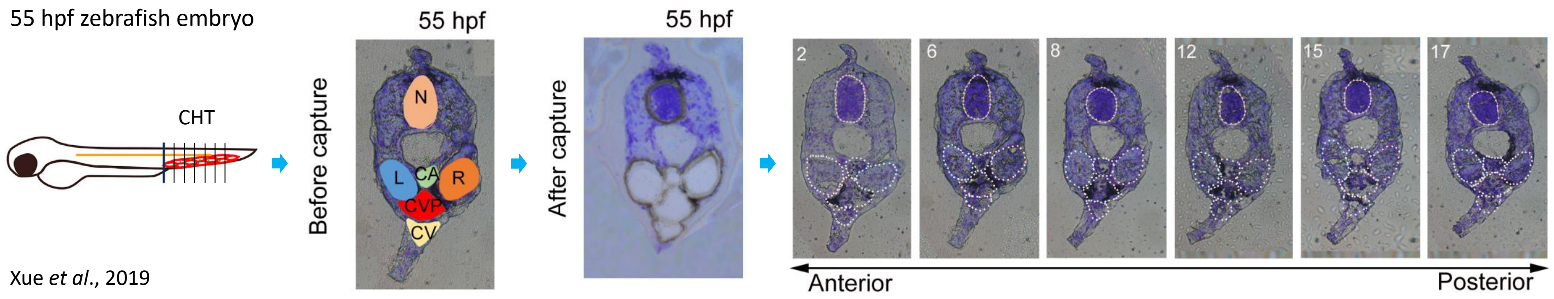

Figure S13. Geo-seq for the zebrafish embryo. Related to Figure 6.

Geo-seq experiment for the caudal hematopoietic tissue (CHT) of 55-hpf zebrafish embryo. The six Geo-seq regions include neuro (N), left muscle (L), right muscle (R), caudal artery (CA), caudal vein (CV) and caudal vein plexus (CVP). Geo-seq images were obtained from (*Xue et al., 2019*).
